# Engineered Mesenchymal Stem Cell-Derived Small Extracellular Vesicles Mitigate Liver Fibrosis by Delivering USP10 to Reprogram Macrophage Phenotype

**DOI:** 10.34133/bmr.0244

**Published:** 2025-08-26

**Authors:** Siyuan Tian, Xia Zhou, Linhua Zheng, Jingyi Liu, Miao Zhang, Shuoyi Ma, Xiaohong Zheng, Guanya Guo, Ruobing Ju, Fangfang Yang, Yansheng Liu, Bo Li, Yinan Hu, Erzhuo Xia, Rui Su, Keshuai Sun, Lina Cui, Changcun Guo, Xinmin Zhou, Jingbo Wang, Yulong Shang, Ying Han

**Affiliations:** ^1^ Xijing Hospital of Digestive Diseases, State Key Laboratory of Holistic Integrative Management of Gastrointestinal Cancers and National Clinical Research Center for Digestive Diseases, Fourth Military Medical University, Xi’an, Shaanxi, China.; ^2^ Department of Gastroenterology, The 989(th) Hospital of the People’s Liberation Army Joint Service Support Force, Luoyang 471031, China.; ^3^ Department of Biochemistry and Molecular Biology, Fourth Military Medical University, Xi’an, China.; ^4^ Department of Radiation Oncology, Xijing Hospital, Fourth Military Medical University, Xi’an, China.; ^5^ Department of Gastroenterology, The Air Force Hospital From Eastern Theater of PLA, Nanjing 210002, Jiangsu, China.

## Abstract

The utilization of mesenchymal stem cells (MSCs) serves as an encouraging strategy for treating liver fibrosis. However, precise mechanisms are not completely understood. Recently, small extracellular vesicles (sEVs) have emerged as major paracrine effectors mediating the anti-fibrotic effects of MSCs. This study seeks to examine the healing properties of MSCs-sEVs on liver fibrosis and decipher the associated signaling pathways. Herein, MSCs substantially ameliorated carbon tetrachloride (CCL4)-induced liver inflammation and fibrosis in mice, with this effect predominantly attributed to their derived sEVs. Both in vivo and in vitro experiments verified that MSCs-sEVs skewed the phenotype of liver macrophages into an anti-fibrotic phenotype. Mass spectrometry analysis showed that ubiquitin-specific peptidase 10 (USP10) was significantly enriched in MSCs-sEVs, which was critical for protection against liver fibrosis. USP10 stabilizes Krüppel-like factor 4 (KLF4) via deubiquitination, participating in the modulation of macrophage phenotypes. Mechanistically, KLF4 reprograms macrophages to enhance their anti-inflammatory and repairing functions by modulating NF-κB/STAT6 signaling and regulating the transcription of MMP12. Finally, the exogenous incorporation of USP10 into MSCs-sEVs via genetic engineering further potentiated their antifibrotic effects. These findings deepen the knowledge regarding the cellular pathways through which MSCs ameliorate liver fibrosis, offering a theoretical basis for sEV-based therapeutic strategies.

## Introduction

Chronic liver disease represent a major worldwide health issue, causing around 2 million fatalities annually, with 1 million attributed to cirrhosis [1]. Liver fibrosis, a frequently observed pathological phenomenon in chronic liver injuries, stems from various causes such as viruses, alcohol consumption, and metabolic disorders. This condition marks an essential stage in the advancement from liver injury toward cirrhosis and ultimately organ failure [2]. So far, therapeutic modalities for liver fibrosis and cirrhosis are restricted and orthotropic liver transplantation remains the sole effective treatment for decompensated cirrhosis. Nevertheless, this option is hampered by the severe shortage of donor organs [3].

Mesenchymal stem cells (MSCs) are recognized as potential candidates for liver disease management, due to their immunomodulatory and tissue repair capabilities [4]. Several clinical studies reported that MSCs infusion markedly improved liver function and long-term survival in individuals with decompensated cirrhosis [5,6]. Animal studies have also confirmed the anti-fibrotic properties of MSCs [7]. However, the widespread clinical application of MSCs has been largely hindered by unclear mechanisms of action. Furthermore, the direct application of MSCs as a cellular therapy face limitations regarding efficacy and safety [8]. Recently, growing evidence has indicated that small extracellular vesicles (sEVs) play a pivotal role in the therapeutic effects of MSCs through the paracrine way. sEVs are lipid bilayer membrane vesicles with diameters ranging from 30 to 150 nm, which are formed by the endocytosis–fusion–efflux process of cell. They play crucial roles in various biological functions, such as maintaining tissue homeostasis, modulating the immune system, and facilitating tissue repair processes, by delivering molecular cargo (DNA, RNA, protein) to recipient cells [9]. As a cell-free therapeutic approach, MSCs-sEVs avoid potential risk of immune rejection, malignant transformation, or undesired differentiation, holding great value for clinical transformation.

Macrophages play a critical role in the initiation, progression, and resolution of liver fibrosis [10]. In the liver, macrophages can be broadly classified as Kupffer cells (KCs) and monocyte-derived macrophages (MDMs). They are highly heterogeneous cells with great functional plasticity, exhibiting either pro- or anti-fibrotic functions [11]. As the first sensors of liver injuries, KCs secrete chemokines to initiate inflammation. Subsequently, both KCs and recruited MDMs differentiate into proinflammatory macrophages, secreting multiple inflammatory and profibrotic cytokines that activate hepatic stellate cells (HSCs), thus promoting liver fibrosis [12]. During the repair process, however, macrophages switch to a reparative phenotype contributing to tissue remodeling by releasing anti-inflammatory cytokines and matrix metalloproteinases (MMPs) [13]. Therefore, targeting macrophage phenotype transformation is a crucial therapeutic approach for liver fibrosis treatment. Our prior research identified that MSCs-sEVs contain diverse biological molecules linked to tissue repair and anti-inflammatory process. Similar to their parental cells, MSCs-sEVs effectively ameliorated carbon tetrachloride (CCL4)-induced liver fibrosis [14–16]. Systemically infused MSCs-sEVs were primarily internalized by the hepatic macrophages [17]. However, the effects of MSCs-sEVs on macrophage phenotype regulation in liver fibrosis and the underlying molecular mechanisms remain to be fully elucidated.

In this study, a CCL4-induced liver fibrosis model was employed to assess the therapeutic potential of MSCs-sEVs. Our findings demonstrate that MSCs-sEVs effectively inhibited inflammatory response and ameliorated liver fibrosis. Both in vitro and in vivo experiments highlighted macrophages as key target cells for the biological actions of MSCs-sEVs. Specifically, MSCs-sEVs were found to induce a phenotypic shift in macrophages, facilitating their transformation from profibrotic toward anti-fibrotic phenotypes. Mass spectrometry (MS) combined with bioinformatics analysis further indicated that ubiquitin-specific peptidase 10 (USP10) plays a vital function in sEV-mediated anti-fibrotic effects. Mechanistically, USP10 deubiquitinates and stabilizes Krüppel-like factor 4 (KLF4) expression, which promotes the anti-inflammatory and reparative properties of macrophages by regulating nuclear factor κB (NF-κB)/signal transducer and activator of transcription (STAT6) signaling and MMP12 expression.

## Materials and Methods

### Animals and experiment groups

Male C57BL/6J mice (8 to 10 weeks old) were obtained from the Experimental Animal Centre of Air Force Military University (Xi’an, China). Mice were kept in a pathogen-free setting with unlimited access to food and water. The Animal Welfare and Ethics Committee of the Air Force Military University approved the animal research protocol (no. 20220465). All experimental procedures adhered to established guidelines for laboratory animal care and use. Liver fibrosis was established in the mice by intraperitoneal administration of 0.2 ml/20 g of a 20% (v/v) solution of CCL4 in olive oil, administered twice weekly for a period of 8 weeks. Following fibrosis induction, the mice were randomly assigned to treatment or control groups, with each group consisting 6 to 8 mice. For treatment groups, 1 × 10^6^ cells or 150 μg of sEVs was diluted in 150 μl of phosphate-buffered saline (PBS) and injected via tail. For the control groups, an equivalent volume of PBS was administered in the same manner. After the injections, the mice continued to receive CCL4 for an additional 2 weeks. Following this period, the animals were euthanized for subsequent tissue sectioning and examination.

### Cell culture and treatment

The human umbilical cord-derived MSCs (hUC-MSCs) for this investigation were procured from Tianjin AmCellGene Engineering Co. Ltd., China. The MSCs underwent cultivation in mesenchymal basal medium (Dakewe Biotech Co. Ltd., China), enhanced with serum-free supplementation. Surface marker identification of MSCs was executed through flow cytometry. Differentiation potential was assessed by culturing MSCs under conditions that induced adipogenic and osteogenic differentiation. Cells in passages 4 to 7 were used in the present study.

RAW264.7 cell line was acquired from the American Type Culture Collection (ATCC) and maintained in Dulbecco’s modified Eagle’s medium (DMEM; Gibco, NY) comprising 10% fetal bovine serum (FBS; Gibco, NY) plus 1% penicillin–streptomycin. Bone marrow-derived macrophages (BMDMs) were isolated from C57BL/6J mice aged 6 to 8 weeks following a previously described protocol [18]. M1 (classical activation: proinflammatory) and M2 (alternative activation: tissue remodeling) macrophages were induced using lipopolysaccharide (LPS) (100 ng/ml), interferon-γ (IFN-γ) (20 ng/ml), or interleukin-4 (IL-4) (20 ng/ml). To investigate the effects of MSCs-sEVs on macrophage phenotype switching, 10 μg/ml MSCs-sEVs were cocultured with macrophages for 12 to 24 h.

Human HSCs (LX2) were purchased from Procell Life Science & Technology Co. Ltd. and cultured according to the standard procedures. For coculture experiments, LX2 cells were cultured with conditioned medium derived from BMDMs treated with or without MSCs-sEVs for 24 h.

### Extracellular vesicle isolation and characterization

Upon MSCs achieving 70% to 80% confluence, EV-depleted medium (Umibio, Shanghai, China) replaced the original culture medium. Following 48 h, the supernatant from cells underwent a series of centrifugation steps: initial spinning at 300*g* for 10 min and subsequent processing at 2,000*g* for 10 min (both maintained at 4 °C) to eliminate cellular components and debris. The obtained solution was processed at 10,000*g* for 30 min to eliminate larger vesicles and contaminants. Additional ultracentrifugation occurred at 100,000*g* for 70 min at 4 °C, with subsequent PBS washing under identical conditions. For morphological characterization, freshly isolated sEVs were loaded onto the copper mesh, stained with phosphotungstic acid for 10 min, and examined using transmission electron microscope. Nanoparticle tracking analysis (NTA) was employed to determine particle size and concentration of isolated sEVs. The profiles of EV markers, including CD9, CD81, and TSG101, were detected by Western blot (WB) analysis, while the endoplasmic reticulum protein calnexin served as a negative control.

### Labeling and uptake of MSCs-sEVs

PBS solution containing extracellular vesicles was incubated with PKH26 or PKH67 dye (Umibio, Shanghai, China) at 37 °C for 30 min, following the supplier’s protocols. Subsequently, BMDMs were cocultured with PKH dye-labeled extracellular vesicles for 12 h, followed by washing with PBS. Confocal microscopy or flow cytometry analysis was applied to detect the cellular uptake of MSCs-sEVs. For in vivo imaging, isolated MSCs-sEVs underwent fluorescent staining utilizing PKH dye or DiR dye markers (Umibio, Shanghai, China) (Invitrogen, USA) according to the manufacturer’s instructions and then intravenously administered to the fibrotic mice. Biodistribution was analyzed 24 h post-administration using the IVIS Spectrum small animal imaging system, manufactured by IVIS Kinetics (Caliper Life Science). Organs were also harvested for ex situ imaging analysis.

### RNA extraction and quantitative real-time polymerase chain reaction evaluation

Total RNA isolation was extracted with TRIzol (Thermo Fisher Scientific, USA), with subsequent reverse transcription executed employing PrimeScript RT Master Mix (RR036A, Takara, Tokyo). The amplification process employed TB Green Premix Ex Taq II on the CFX96 Real-Time System (Bio-Rad, CA). Table S1 lists the specific primers for each transcript, with β-actin mRNA serving as the internal control for normalizing mRNA expression.

### WB analysis

Protein samples underwent extraction and quantification procedures. A sum of 30 μg of protein underwent separation via sodium dodecyl sulfate–polyacrylamide gel electrophoresis (SDS-PAGE) gels and subsequently moved into nitrocellulose membranes (Bio-Rad Biotechnology, USA). The membrane samples underwent blocking treatment for 60 min with Tris-buffered saline Tween-20 (TBST) buffer plus 5% skim milk to minimize nonspecific binding events. The specimens were subjected to overnight reaction with specific primary antibodies at 4 °C following the blocking process. Following this step, the membranes received 3 TBST washing cycles to eliminate excess unbound antibodies, succeeded by incubation with peroxidase-linked secondary antibodies. The protein signals were detected through enhanced chemiluminescence methodology. The selected primary antibodies encompassed anti-collagen І (ab138492, Abcam), anti-α-smooth muscle actin (α-SMA) [19245, Cell Signaling Technology (CST)], anti-β-actin (66009-1-Ig, Proteintech), anti-CD9 (ab223052, Abcam), anti-CD81 (ab109201, Abcam), anti-TSG101 (ab125011, Abcam), anti-calnexin (10427-2-AP, Proteintech), anti-inducible nitric oxide synthase (iNOS) (ab15323, Abcam), anti-Arg1 (ab64693, Abcam), anti-MMP12 (22989-1-AP, Proteintech), anti-P65 (8242T, CST), anti-p-P65 (3033T, CST), anti-STAT6 (51073-1-AP, Proteintech), anti-p-STAT6 (ab263947, Abcam), anti-USP10 (19374-1-AP, Proteintech), and anti-KLF4 (sc-393462, Santa Cruz Biotechnology; 11880-1-AP, Proteintech).

### Biochemical analysis and histological staining

At the designated time point, serum samples were collected and measured using an automated biochemistry analyzer. Liver tissue samples were processed, paraffin-embedded, and cut into 5-μm sections utilizing a microtome. The tissue sections experienced dehydration through graduated ethanol concentrations followed by xylene clearing. Routine histological examination was conducted utilizing hematoxylin and eosin (H&E) staining, along with Sirius red staining for fibrosis assessment.

### Immunohistochemistry staining

The paraffin-embedded liver tissue underwent dewaxing by immersing it in xylene, succeeded by rehydration in a gradient concentration of ethanol solution. Antigen retrieval was then conducted through microwave treatment utilizing sodium citrate buffer as the retrieval agent. To block endogenous peroxidase activity, a solution containing 1% H_2_O_2_ was applied. Subsequently, after blocking nonspecific binding sites for antibodies using goat serum for 30 min, the tissue slices were maintained at 4 °C overnight with anti-α-SMA. Following thorough washing steps using PBS, the specimens underwent secondary antibody treatment for 60 min and subsequently visualized via diaminobenzidine (DAB) staining.

### Immunofluorescent staining

BMDMs underwent washing with cold PBS and followed by fixation using 4% paraformaldehyde. Cell permeabilization was achieved utilizing 0.1% Triton X-100 solution for 7 min. Following permeabilization, the BMDMs underwent incubation with mouse anti-KLF4 and rabbit anti-USP10 antibodies to visualize the colocalization using confocal microscopy.

The tissue sections underwent preparation and overnight exposure at 4 °C to primary antibodies, specifically anti-α-SMA (19245, CST) diluted 1:500, anti-collagen І (ab138492, Abcam) diluted 1:200, anti-F4/80 antibody (ab6640, Abcam) diluted 1:100, anti-iNOS antibody (ab15323, Abcam) diluted 1:100, or anti-CD206 antibody (ab64693, Abcam) diluted 1:100. Following 3 PBS washes, the specimens received secondary antibody and 4′,6-diamidino-2-phenylindole (DAPI) staining, followed by examination under a fluorescence microscope.

### Isolation of liver nonparenchymal cells

Liver nonparenchymal cells underwent separation by perfusing and digesting the liver with collagenase IV (Sigma) and 30% Percoll (GE Healthcare), as per established protocols [19]. Hepatic macrophages were purified by isolating hepatic nonparenchymal cells (NPCs) using a 50%/25% Percoll gradient centrifugation at 1,800*g* for 15 min. The middle layer containing NPCs was procured and rinsed for subsequent WB or quantitative real-time polymerase chain reaction (qRT-PCR) analysis.

### Flow cytometry analysis

Single-cell suspensions derived from hepatic NPCs underwent treatment with anti-mouse FcR blocking reagent and were labeled with combined fluorescence-conjugated antibodies [BV510-Zombie, allophycocyanin (APC)/cyanine 7 anti-mouse CD45.2, phycoerythrin (PE) anti-mouse F4/80, fluorescein isothiocyanate (FITC) anti-mouse/human CD11b, peridinin chlorophyll protein (PerCP)/cyanine 5.5 anti-mouse CD86, and APC anti-mouse CD206 (MMR)]. Flow cytometric measurements were procured utilizing BD FACSCanto II, succeeded by data analysis through FlowJo software.

### Plasmid and siRNA transfection

The USP10 plasmid, KLF4 plasmid, and control plasmid were constructed by TSINGKE Biological Technology and introduced temporarily into specified cells utilizing jetPRIME DNA transfection reagents (Polyplus, France). Small interfering RNAs (siRNAs) targeting USP10, KLF4, and a scrambled control were produced by Sangon Biotech Co. Ltd. (Shanghai, China) and transfected using Lipofectamine RNAiMax reagent (Invitrogen). The effects of gene silencing and overexpression were assessed 24 h post-transfection via qRT-PCR and 48 h post-transfection via WB analysis.

### Lentivirus construction and transfection

Lentiviral vectors containing shNC [negative control (NC)] and shUSP10 were purchased from Hanheng Biotechnology. Overexpression lentiviruses for USP10 as well as control lentiviruses were obtained from Genechem Biotechnology. When the cell density reached 30% to 50% confluence, MSCs underwent lentiviral transduction at a multiplicity of infection of 50. After a cultivation interval of 6 to 8 h, the transfection medium was exchanged for fresh, complete medium. Lentiviral interference efficiency was confirmed using WB 72 h post-transfection.

### Coimmunoprecipitation

Cellular extracts underwent preliminary clearing with Protein G beads during a 1-h period, followed by overnight binding with target antibodies conjugated to A/G-agarose beads while rotating horizontally at 4 °C. Finally, the immunocomplexes were rinsed utilizing chilled lysis buffer and eluted by boiling before being subjected to WB analysis.

### Luciferase reporter assays

Full-length dual-luciferase reporter gene plasmids containing the MMP12 promoter as well as dual-luciferase reporter gene plasmids with mutations in putative KLF4 binding sites were generated. Subsequently, these constructed plasmids were cotransfected into human embryonic kidney 293 cells along with pcDNA3.1-KLF4 plasmid or negative control (NC). Renilla luciferase reporter plasmids were transfected for internal normalization. Forty-eight hours after the transfection process, the relative luciferase activity was evaluated employing the Dual-Glo Luciferase Assay Kit provided by Promega.

### Chromatin immunoprecipitation

RAW264.7 cells were exposed to 1% formaldehyde at room temperature for a duration of 10 min to induce cross-linking, after which the reaction was quenched using glycine. Chromatin immunoprecipitation (ChIP) analysis utilized a Pierce Agarose ChIP Kit (Thermo USA, catalog no. 26156). From the sonicated cell lysates, DNA was immunoprecipitated using either anti-KLF4 antibodies (ab215036, Abcam) or anti-IgG antibodies as a negative control. Subsequently, the immunoprecipitation-derived purified DNA/protein complexes underwent quantitative real-time PCR (qRT-PCR) analysis to amplify KLF4-specific binding sites. The primer sequences employed in this process are depicted in Table S2.

### RNA sequencing and MS proteomics analysis

RNA isolation, library construction, deep sequencing, and annotation procedures were carried out by Gene Denovo Technology Co. Ltd. (Guangzhou, China). Gene expression levels were quantified utilizing fragments per kilobase of transcript per million fragments mapped (FPKM). Differential gene expression between groups was analyzed utilizing the edgeR software with a false discovery rate (FDR) < 0.05 and |logFC| ≥ 1 as the threshold. Gene Ontology (GO) and Kyoto Encyclopedia of Genes and Genomes (KEGG) analyses were conducted to functionally annotate these differentially expressed genes.

Proteins from MSCs or MSCs-sEVs were collected for Q-Exactive MS following the standard protocol previously described [20]. The gel slices were visualized by Coomassie blue staining and cut for MS analysis, which was conducted using the Q-Exactive system (Thermo Fisher Scientific, USA) in our laboratory. Protein identification and quantitation were conducted using Proteome Discoverer software (v1.4; Thermo Fisher Scientific, USA). Proteins exhibiting differential expression were identified using a threshold of a 2.0-fold change and a *P* value below 0.05. Gene expression patterns were illustrated using volcano plots and heatmaps, employing the R packages “ggplot” and “pheatmap”, respectively.

### Bioinformatics analysis

The single-cell transcriptome dataset GSE110746 and transcriptome dataset GSE139062 were retrieved from the Gene Expression Omnibus (GEO) database. The cloud-based OmicShare platform (https://www.omicshare.com/) was utilized for dimensional reduction, clustering, and analysis of the GSE110746 dataset. In the GSE139602 dataset, USP10 expression was observed at various stages of liver disease. At the cellular level, the expression of USP10 was analyzed using the Human Protein Atlas (HPA) database. A combination of GeneCards database and UbiBrowser 2.0 was applied to predict potential substrates of USP10. Correlation analysis of the KLF4 and MMP12 level in liver tissues was evaluated through the Gene Expression Profiling Interactive Analysis (GEPIA) database.

### Statistical analysis

The mean ± SD was utilized to present the data. For sample comparison, the Student’s *t* test was applied for 2 groups and one-way analysis of variance (ANOVA) was utilized for multiple groups. Spearman correlation coefficient analysis was employed to examine the linear relationship between KLF4 and MMP12. Values of *P* < 0.05 indicated statistical significance. Statistical analyses were executed utilizing GraphPad Prism 8 (GraphPad Inc., San Diego, CA, USA).

## Results

### GW4869 pretreatment impaired anti-fibrotic effects of MSCs

Surface markers and differentiation potential were utilized to characterize the phenotype of MSCs. Flow cytometry revealed that MSCs exhibited high CD29, CD73, CD90, and CD105 levels, with minimal expression of hematopoietic lineage markers like CD31, CD34, and CD45 (Fig. S1A). Under light microscopy, MSCs were spindle-shaped. Under differentiation-inducing conditions, MSCs showed osteogenic or adipogenic differentiation ability, as shown in Fig. S1B. These results showed that MSCs were successfully isolated and could be used for subsequent experiments.

To further demonstrate the role of paracrine factors, especially sEVs, on anti-fibrotic effects of MSCs, sEV inhibitor GW4869 was used for the pretreatment of MSCs before transplantation. Therapeutic effects of different groups were evaluated 2 weeks post-MSC injection (Fig. S1C). Histological staining results showed that MSCs significantly reduced hepatic inflammation and fibrosis compared to the PBS group. In contrast, GW4869 pretreatment impaired these effects, leading to failure of transplanted MSCs to regulate inflammation response and collagen degradation (Fig. S1D). Compared to the MSC therapy group, qRT-PCR and WB analysis also confirmed that fibrotic markers were significantly higher in the livers from GW4869-pretreated group (Fig. S1E to G).

### MSC-sEV administration attenuated liver fibrosis

**Fig. 1.**
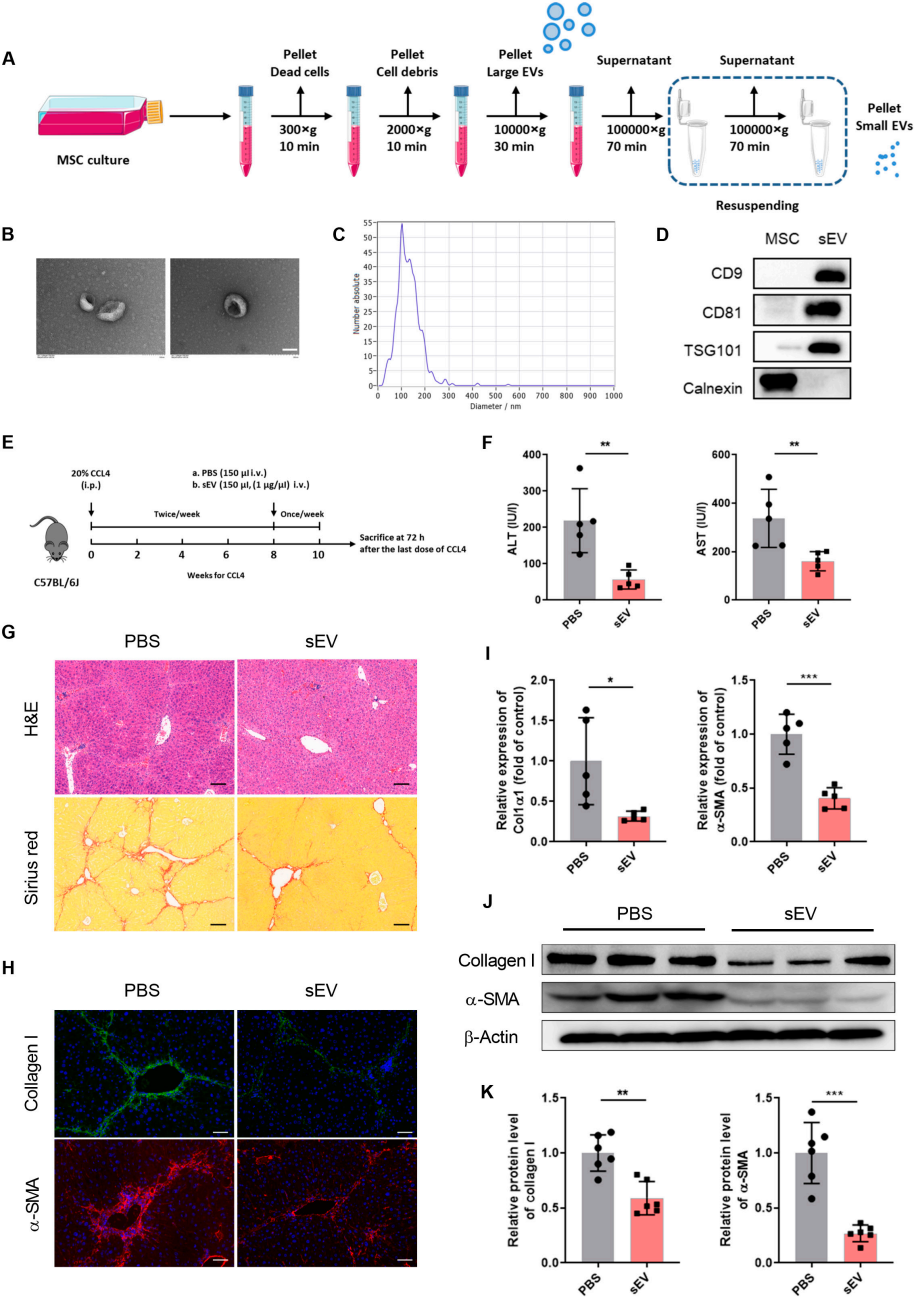
Therapeutic efficacy of MSCs-sEVs in the CCL4-induced murine liver fibrosis model. (A) Schematic diagram of extracellular vesicle isolation protocol. (B) Representative TEM images of MSCs-sEVs. (C) Particle size distribution of MSCs-sEVs as detected by NTA. (D) WB analysis of MSC-sEV markers and negative control. (E) Schematic illustration of the treatment schedule. (F) Evaluation of liver function indicators following MSC-sEV treatment. (G) Representative images of H&E and Sirius red staining of liver sections. Scale bar, 100 μm. (H) Immunofluorescence staining of collagen I and α-SMA in liver tissues. Scale bar, 50 μm. (I) Relative mRNA expression of col1α1 and α-SMA in liver tissues, quantified by qRT-PCR. (J and K) Protein expression of collagen I and α-SMA in liver tissues, analyzed by WB assay. Data are presented as mean ± SD, *n* = 5 to 6 per group; *P < 0.05, **P < 0.01, ***P < 0.001.

The acquired findings suggested that sEV could facilitate the therapeutic impact of MSCs regarding liver inflammation and fibrotic progression. Next, to further validate its direct action, we collected culture supernatant of MSCs and extracted MSCs-sEVs by ultracentrifugation (Fig. 1A). The isolated particles were characterized based on their morphology, size, and specific markers. As shown in Fig. 1B, transmission electron microscopy (TEM) results showed that MSCs-sEVs presented a typical biconcave disc-shaped morphology. The particle size distribution was determined by NTA, ranging mainly at about 100 nm (Fig. 1C). Additionally, WB analysis further confirmed that MSCs-sEVs positively expressed CD9, CD81, and TSG101, but negatively expressed calnexin (Fig. 1D).

The therapeutic efficacy of MSCs-sEVs was evaluated through tail vein injection of either MSCs-sEVs or PBS in fibrotic mice (Fig. 1E). Compared to the control group, liver function indicators like alanine aminotransferase (ALT) and aspartate aminotransferase (AST) were markedly decreased after MSC-sEV treatment (Fig. 1F). Histological examination revealed that MSCs-sEVs substantially reduced the infiltration of inflammatory cells and improved liver tissue structure, as well as collagen deposition, as evidenced by H&E and Sirius red staining (Fig. 1G). Immunofluorescence staining suggested that fibrosis markers, collagen I and α-SMA levels, were both diminished in the MSC-sEV transplantation group (Fig. 1H). In addition, we utilized qRT-PCR and WB to examine the mRNA and protein levels of these fibrotic markers in liver tissues. The corresponding gene expression changes could be corroborated with the above results, as indicated in Fig. 1I to K. Collectively, these findings highlight the critical role of sEVs in mediating MSCs’ anti-fibrotic effects.

### Macrophages are pivotal for the therapeutic action of MSCs-sEVs

**Fig. 2.**
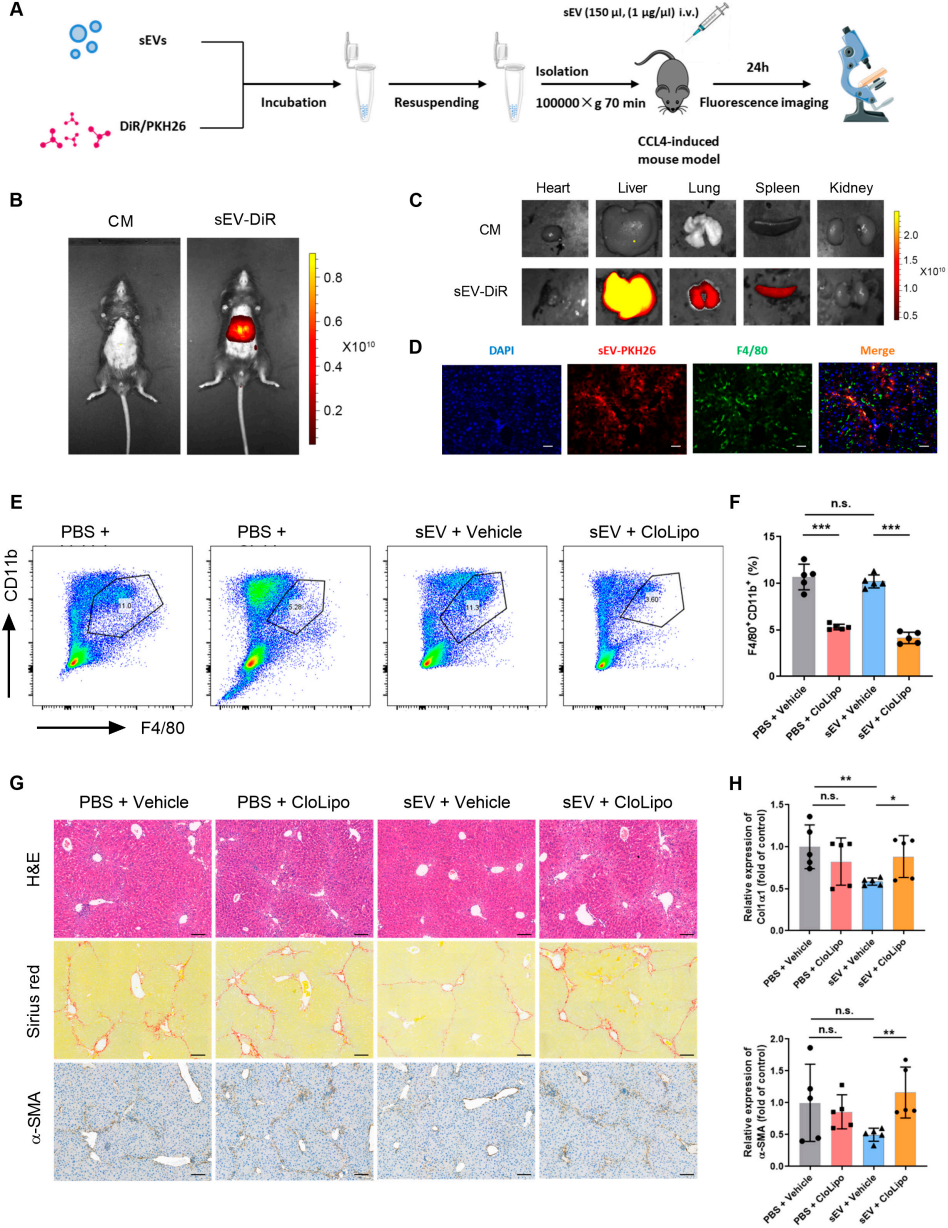
Macrophages are key mediators for MSCs-sEVs to exert therapeutic effects. (A) Experimental design for tracking the distribution of DiR- or PKH26-labeled MSCs-sEVs in fibrotic mice. (B and C) Fluorescence signal of different organs was detected by using an in vivo imaging system. CM, conditioned medium. (D) Uptake of PKH26-labeled (red) MSCs-sEVs by macrophages (F4/80, green) in the liver. Scale bar, 50 μm. (E) Representative flow cytometry results showing macrophage depletion efficiency. (F) Statistical analyses of the percent of F4/80^+^CD11b^+^ cells in each group. (G) Representative liver sections stained with H&E, Sirius red, and α-SMA from different groups. Scale bar, 100 μm. (H) Relative mRNA expression of col1α1 and α-SMA in liver tissues, quantified by qRT-PCR. Data are denoted as mean ± SD, *n* = 5 per group; *P < 0.05, **P < 0.01, ***P < 0.001, n.s., no significance.

Following the elucidation of the therapeutic effects of MSCs-sEVs, we further explored potential targets of MSCs-sEVs for treating liver fibrosis. We visualized their distribution in vivo by using fluorescent dye DiR or PKH26 (Fig. 2A). Results of liver imaging analyses showed that the fluorescence signal was strongly detected in the liver, followed by the spleen and lung (Fig. 2B and C). It is well known that macrophages are abundant in the liver. During liver injury, large amounts of MDMs are recruited to the liver, amplifying the inflammatory response. Therefore, we examined whether MSCs-sEVs could be taken up by macrophages in the liver. Colocalization of F4/80 (macrophage marker) with MSCs-sEVs was observed by dual immunofluorescence assays (Fig. 2D). Above-described results suggested the possible interactions between administrated MSCs-sEVs and macrophages in the context of liver fibrosis. Next, macrophage depletion experiments were conducted to ascertain the function of macrophages in the process of liver repair mediated via MSCs-sEVs. Depletion efficiency was quantified using flow cytometry. The statistical results revealed a significant reduction of hepatic macrophages than those in control groups (Fig. 2E and F). Compared to the PBS + Vehicle group, alleviated inflammation and reduced fibrosis were observed in the sEV + Vehicle group. However, macrophage depletion notably compromised the antifibrotic effects of MSCs-sEVs, as shown by elevated fiber deposition and expression of fibrosis markers relative to the sEV + Vehicle group (Fig. 2G and H). All these data reflect the importance and necessity of macrophages for MSCs-sEVs to exert therapeutic effects.

### MSCs-sEVs induced the macrophage phenotype reprogram in fibrotic liver

The above results suggested that macrophages are important target cells for MSCs-sEVs against liver fibrosis. For in-depth characterization of functional phenotypes of macrophages after MSC-sEV treatment, we mined a publicly available single-cell RNA sequencing dataset (GSE168042). Single cells from the livers of PBS-, MSC-, or sEV-treated fibrotic mice were pooled for this analysis. Dimensionality reduction analysis [t-distributed stochastic neighbor embedding (t-SNE)] segregated all these cells into 11 clusters per their gene expression pattern (Fig. S2A). On this basis, we next analyzed the different macrophage subsets in detail. These cells could be further divided into 4 clusters (Fig. S2B). Expression of classical pro- and anti-inflammatory marker as well as matrix metalloproteins (MMPs) was depicted in a heatmap. As shown in Fig. S2C, cluster 2 highly expressed pro-inflammatory markers CD80 and CD86, while cluster 3 highly expressed the anti-inflammatory marker MRC1 (CD206). Of all MMPs, MMP12 and MMP13 exhibited higher expression levels and mainly distributed in clusters 1 and 3. We calculated the composition ratio of 4 clusters in different treatment groups. MSCs-sEVs significantly reduced the proportion of cluster 2, whereas there was a marked increase in the proportion of cluster 3 (Fig. S2D).

**Fig. 3.**
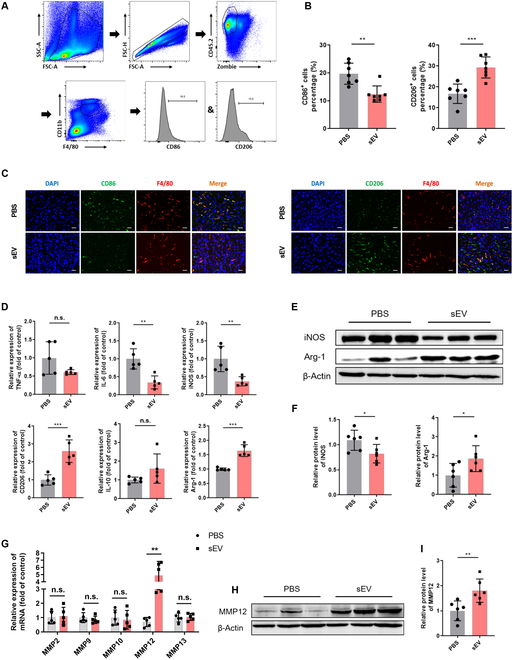
Engrafted MSCs-sEVs reprogrammed macrophage phenotype in CCL4-induced liver fibrosis model. (A) Gating strategy and representative flow cytometry plots. (B) Proportion of CD86^+^ or CD206^+^ macrophages in fibrotic livers post-MSC-sEV transplantation, as determined by flow cytometry (FCM) analysis. (C) Representative immunofluorescence images of CD86^+^ (green) or CD206^+^ (green) macrophages in each group. Scale bar, 50 μm. (D) Relative mRNA expression of pro- or anti-inflammatory genes in liver tissues. (E and F) iNOS and Arg1 protein expression was examined by WB. (G) Relative mRNA expression of MMPs in liver tissues was quantified by qRT-PCR. (H and I) MMP12 protein expression was analyzed by WB. Data are presented as mean ± SD, *n* = 5 to 7 per group; *P < 0.05, **P < 0.01, ***P < 0.001, n.s., no significance.

Above bioinformatics analysis provided a hint that MSCs-sEVs could reshape macrophage phenotypes to promote their anti-inflammatory and repairing properties. To further verify the findings mentioned above, the proportion of pro-inflammatory (CD86^+^) and anti-inflammatory (CD206^+^) phenotype cells was examined by flow cytometry. The specific gating strategy is depicted in Fig. 3A. Statistical results showed that there were a higher proportion of reparative macrophages following MSC-sEV treatment, as compared with more adverse inflammatory macrophages in the PBS-treated controls (Fig. 3B). Immunofluorescence staining was consistent with these results (Fig. 3C). In addition, we examined the expression of other pro- or anti-inflammatory markers. IL-6 and iNOS showed obvious down-regulation, whereas CD206 and Arg1 were up-regulated (Fig. 3D to F). The expression of MMPs was also examined correspondingly. Only MMP12 expression was significantly enhanced following MSC-sEV treatment at the mRNA and protein level (Fig. 3G to I).

### MSCs-sEVs modulated the polarization of macrophages and increased MMP12 expression in vitro

**Fig. 4.**
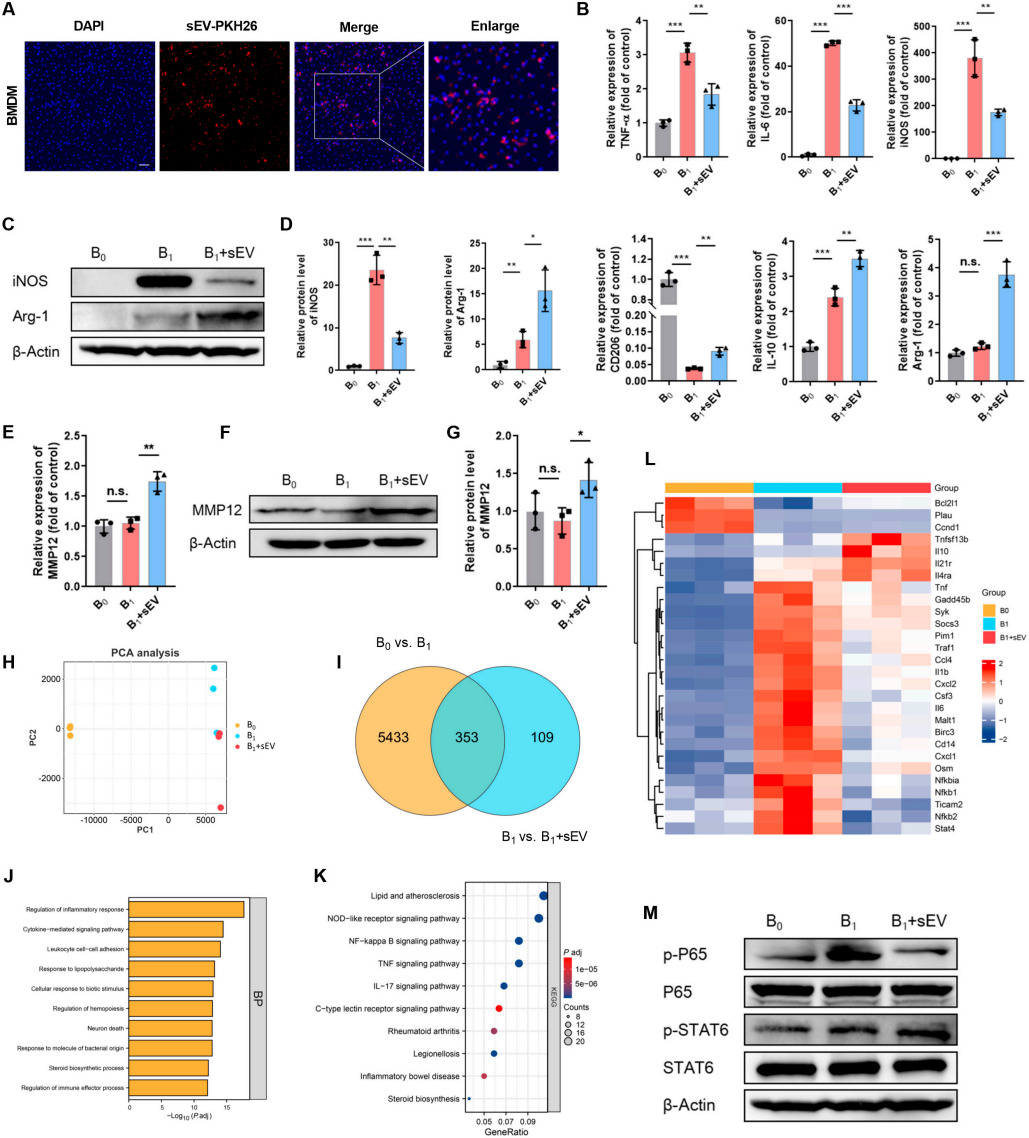
MSCs-sEVs regulated macrophage phenotype switch and MMP12 expression in vitro. (A) Representative fluorescence image of MSCs-sEVs uptake in BMDMs (red, PKH26-labeled MSCs-sEV; blue, DAPI). Scale bar, 50 μm. (B) mRNA levels of M1 macrophage markers (TNF-α, IL-6, and iNOS) and M2 macrophage markers (CD206, IL-10, and Arg1), analyzed by qRT-PCR. (C and D) WB analysis for protein expressions of iNOS and Arg1 in BMDMs. (E to G) mRNA and protein levels of MMP12 detected in BMDMs. (H) PCA of the 3 groups. (I) Venn diagram showing the intersection of differentially expressed genes (FDR < 0.05 and |logFC| ≥ 1). (J) Enrichment analysis of biological process. (K) Top 10 significantly enriched pathways from KEGG analysis. (L) Heatmap for the differentially expressed genes involved in the NF-κB and JAK-STAT signaling pathways. (M) Protein level of p65/P65 and p-STAT6/STAT6 was determined by WB. Data are presented as mean ± SD, *n* = 3; *P < 0.05, **P < 0.01, ***P < 0.001, n.s., no significance.

Next, we conducted in vitro experiments on isolated BMDMs to validate the above in vivo results. To monitor the up-take of MSCs-sEVs at a cellular level, BMDMs were cocultured with PKH26-labeled MSCs-sEVs. As revealed by immunofluorescence, notable red fluorescence signal was detected in the cytoplasm of BMDMs incubated with MSCs-sEVs following 12 h (Fig. 4A). To confirm the regulatory effects of MSCs-EVs on BMDMs, we also treated LPS- and IFN-γ-stimulated BMDMs with MSCs-sEVs. qRT-PCR analysis revealed that MSCs-sEVs suppressed the expression of M1 markers [tumor necrosis factor-α (TNF-α), IL-6, and iNOS] and elevated the expression of the M2 markers (CD206, IL-10, and Arg1) (Fig. 4B). Meanwhile, the alterations in protein expression of iNOS and Arg1 were verified by WB as indicated (Fig. 4C and D). In addition, the regulation of MSCs-sEVs on MMP12 expression was demonstrated at the mRNA and protein level utilizing qRT-PCR and WB, respectively (Fig. 4E to G).

To elucidate the mechanism by which MSCs-sEVs regulate macrophage phenotype switching, RNA sequencing was performed on samples from 3 groups of BMDMs cultured alone (B_0_), stimulated with LPS/IFN-γ (B_1_), or combined exposure to LPS/IFN-γ and MSCs-sEVs (B_1_ + sEV). Principal components analysis (PCA) displayed pronounced difference in the transcriptional profiles among the 3 groups (Fig. 4H). Then, differential gene intersection was obtained by comparing B_1_ individually with the other 2 groups (Fig. 4I). For these 353 common genes, we performed functional enrichment analysis, as depicted in Fig. 4J and K. The results showed that the enriched items were closely linked to the regulation of inflammatory signals, including NF-κB signaling pathway, supporting our observations that MSCs-sEVs inhibited M1 phenotype of macrophages. In addition, Janus kinase (JAK)–STAT signaling pathway, which is closely linked to M2 polarization of macrophages, was also statistically significant in the enrichment results of signaling pathways (data not shown). The expression patterns of genes distributed on the NF-κB signaling pathway and JAK-STAT signaling pathway were depicted in Fig. 4L. Furthermore, the abovementioned signaling pathways were also verified by WB, and the outcomes displayed that MSC-sEV treatment attenuated the phosphorylation of P65 and enhanced STAT6 phosphorylation in inflammatory BMDMs (Fig. 4M).

In liver fibrosis, HSC activation is recognized as a pivotal event. Thus, we further investigated the interaction between MSC-sEV-induced macrophages and HSC activation. As shown in Fig. S3A, LX2 cells were incubated with supernatants from inflammatory BMDM treated with or without MSCs-sEVs. Compared to the PBS group, MSC-sEV treatment significantly reduced the activation effect of BMDM supernatant on LX2, resulting in a marked decrease in fibrosis markers, including collagen I and α-SMA (Fig. S3B to D).

### USP10 is a candidate effector of MSC-sEV-mediated therapeutic effects

**Fig. 5.**
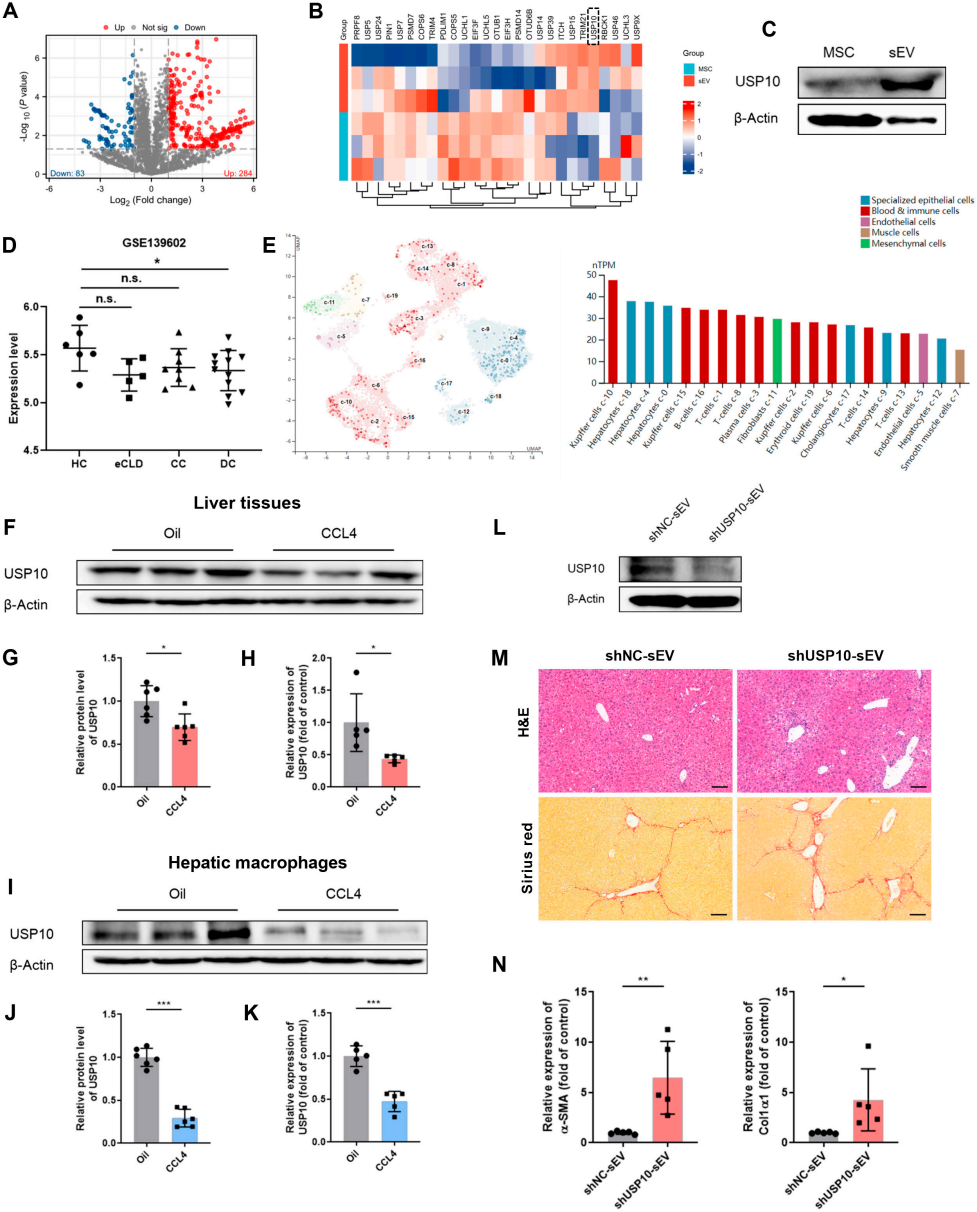
USP10 functions as a hub effector of MSC-sEV-mediated anti-fibrotic effects. (A) Volcano plot presenting proteins differentially expressed in MSCs versus MSCs-sEVs. |logFC| ≥ 1 and *P* < 0.05. (B) Hierarchical cluster of ubiquitin-related protein expression profiles in MSCs and MSCs-sEVs. (C) USP10 protein level analyzed by WB. (D) Transcriptional expression levels of USP10 in liver tissues from healthy controls (HC), patients with chronic liver disease (eCLD), and patients with cirrhosis (CC, compensated cirrhosis; DC, decompensated cirrhosis) (GEO database, GSE139602). (E) Cell type-specific mRNA expression of USP10 in various cells (HPA database). (F to H) USP10 expression levels in liver tissues from control and fibrotic mice were ascertained by WB and qRT-PCR. (I to K) USP10 expression levels in hepatic macrophages from control and fibrotic mice, analyzed by WB and qRT-PCR. (L) USP10 level was analyzed by WB to demonstrate the knockdown efficiency. (M) Representative images of H&E and Sirius red staining of liver sections from the mice in the shNC-sEV and shUSP10-sEV groups. Scale bar, 100 μm. (N) Relative mRNA expression of col1α1 and α-SMA in liver tissues quantified by qRT-PCR. Data are presented as mean ± SD, *n* = 5 to 6 per group; *P < 0.05, **P < 0.01, ***P < 0.001, n.s., no significance.

Encapsulated proteins are one of the key molecules in sEVs that regulate the biological process of recipient cells. To identify the components responsible for the antifibrotic effects, we performed a proteomic analysis for MSCs-sEVs by using MS analysis. Volcano plot identified 284 up-regulated proteins and 83 down-regulated proteins of MSC-sEV samples compared to MSCs (Fig. 5A). Notably, multiple ubiquitin-related proteins were detected in MSCs-sEVs (Fig. 5B). Previous investigations have pointed out that ubiquitination is as crucial mechanism in the regulation of innate immune signaling cascades [21]. It was observed that USP10 was the most enriched in the MSCs-sEVs among these proteins. We also confirmed the expression of USP10 in the MSCs-sEVs by using WB (Fig. 5C). In light of the abundance of USP10 in MSCs-sEVs and regulatory effects of MSCs-sEVs on macrophage phenotype, we therefore focused on the function of USP10 in MSC-sEV-mediated anti-fibrotic effects.

We firstly characterized the expression pattern of USP10 in normal or fibrotic livers by data mining. The GSE139602 dataset was retrieved from the GEO database, which contained liver tissue RNA sequencing data of 6 healthy controls, 5 patients with liver fibrosis, 8 patients with compensated cirrhosis, and 12 patients with decompensated cirrhosis. As shown in Fig. 5D, USP10 expression was significantly down-regulated in liver tissues of patients with cirrhosis compared with healthy controls. The expression of USP10 in human normal liver tissues was further determined at the cellular level using HPA database. The results suggest that USP10 is highly expressed in liver macrophages and hepatocytes (Fig. 5E). Next, we sought to confirm these above findings in animal models. As expected, the USP10 level in liver tissue was notably down-regulated (Fig. 5F to H). To rule out the interference of hepatocytes, isolated liver macrophages underwent USP10 expression analysis, and the results indicated that USP10 expression patterns paralleled those observed in liver tissue samples (Fig. 5I to K).

To further elucidate the role of USP10 in MSC-sEV-mediated alleviation of liver fibrosis, we used lentiviral short hairpin RNA (shRNA) to knock down USP10 in MSCs and harvested sEVs from the cell supernatants. The knockdown efficiency was confirmed by WB (Fig. 5L). These USP10-knockdown extracellular vesicles (shUSP10-sEV) were then administered intravenously via tail vein into fibrotic mice. Histological staining of livers was used to present the pathological changes. H&E staining results suggested that infiltration of inflammatory cells in the shUSP10-sEV group was notably elevated versus that in the shNC-sEV group. Sirius red staining displayed that collagen deposition was markedly elevated in the shUSP10-sEV group (Fig. 5M). The findings aligned with the qRT-PCR detection analyses (Fig. 5N). In in vitro experiments, we also noted that USP10 knockdown impaired the ability of MSCs-sEVs to suppress inflammation and promote MMP12 expression (Fig. S4A and B). Collectively, these results demonstrate that USP10 plays an indispensable role for the therapeutic effects of MSCs-sEVs.

### USP10 interacts with KLF4 and stabilizes its expression

**Fig. 6.**
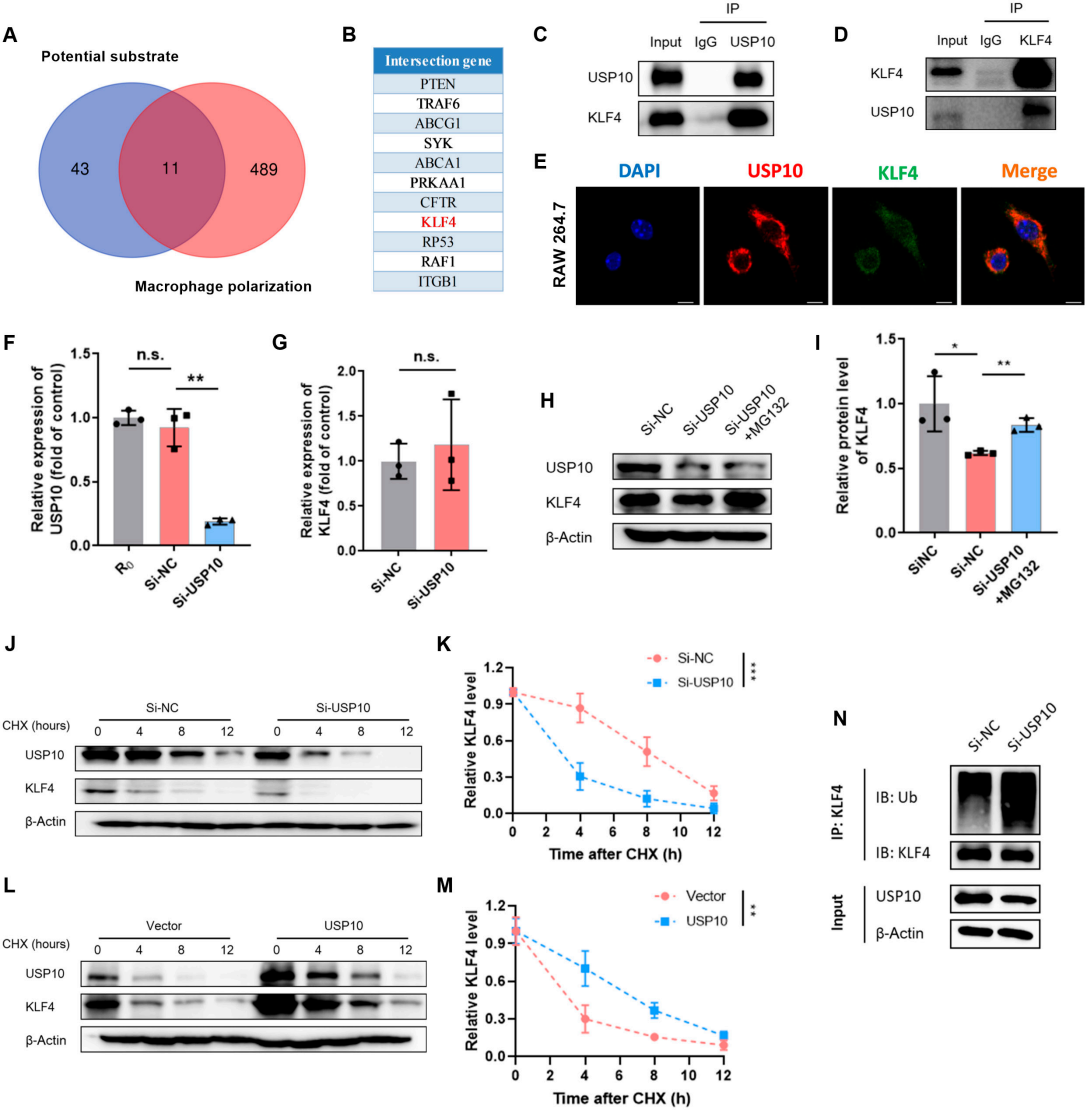
USP10 interacts with and deubiquitylates KLF4. (A and B) Venn diagram showing the intersection between predicted substrates for USP10 (UbiBrowser 2.0 database) and macrophage polarization-related genes (GeneCards database). (C and D) Interaction of endogenous proteins was examined by coimmunoprecipitation assay of USP10 and KLF4 proteins in RAW264.7 lysates. (E) Colocalization of USP10 (red) and KLF4 (green) by immunofluorescence staining in RAW264.7 cells, counterstained with DAPI. Scale bar, 10 μm. (F) Relative mRNA expression of USP10 ascertained by qRT-PCR to evaluate siRNA silencing efficiency in RAW264.7 cells. (G) USP10 mRNA level in RAW264.7 cells transfected with control siRNA or USP10 siRNA. (H and I) Protein expression of USP10 and KLF4 was analyzed by WB in RAW264.7 cells transfected with control siRNA or USP10 siRNA followed by treatment with the proteasome inhibitor MG132 for 8 h. (J and K) KLF4 protein level in RAW264.7 cells transfected with control siRNA or USP10 siRNA was determined by WB in the presence of cycloheximide (CHX; 10 μg/ml) for the indicated time point. (L and M) KLF4 protein level in RAW264.7 cells transfected with empty vector control or USP10 expression plasmid was determined by WB in the presence of CHX (10 μg/ml) for the indicated time point. (N) Knockdown of endogenous USP10 increased the ubiquitination of KLF4 in RAW264.7 cells. Data are denoted as mean ± SD, *n* = 3; *P < 0.05, **P < 0.01, ***P < 0.001, n.s., no significance.

USP10 is one of the major members of the deubiquitinase family. To identify candidate molecules that interact with USP10, UbiBrowser 2.0 database was employed to ascertain the potential substrates, including 34 known and top 20 predicted proteins. Macrophage polarization-related genes were retrieved from GeneCards database, and top 500 genes were screened based on the relevance score, which was then intersected with the above 54 candidate substrates (Fig. 6A). The specific results are shown in Fig. 6B. Among the 11 candidate molecules, the transcription factor KLF4 is a key regulator of macrophage polarization. In RAW264.7 cells, we also confirmed the interaction between USP10 and KLF4 by performing coimmunoprecipitation experiments and immunofluorescence double staining (Fig. 6C to E).

Following the identification of the interaction between USP10 and KLF4, subsequent experiments were conducted to examine the impact of USP10 knockdown on endogenous KLF4 expression in RAW264.7 cells. The finding revealed that silencing USP10 significantly reduced KLF4 protein expression, but not mRNA expression, indicating that USP10 regulates KLF4 expression at the protein level but not at the mRNA level. Meanwhile, we observed that the inhibitory effect of USP10 on KLF4 was reversed by the proteasome inhibitor MG132 (Fig. 6F to I). The effect of USP10 on the stability of KLF4 protein was also monitored. In the presence of the protein synthesis inhibitor cycloheximide, silencing USP10 accelerated KLF4 degradation compared with the control group, whereas overexpression of USP10 markedly inhibited KLF4 degradation (Fig. 6J to M). To investigate whether USP10 directly catalyzes KLF4 deubiquitination, we further employed coimmunoprecipitation assay to quantitatively analyze ubiquitinated KLF4. In RAW264.7 cells, silencing USP10 dramatically increased the ubiquitination of KLF4 (Fig. 6N).

### USP10 regulated inflammatory signals and MMP12 expression in macrophages by stabilizing KLF4

**Fig. 7.**
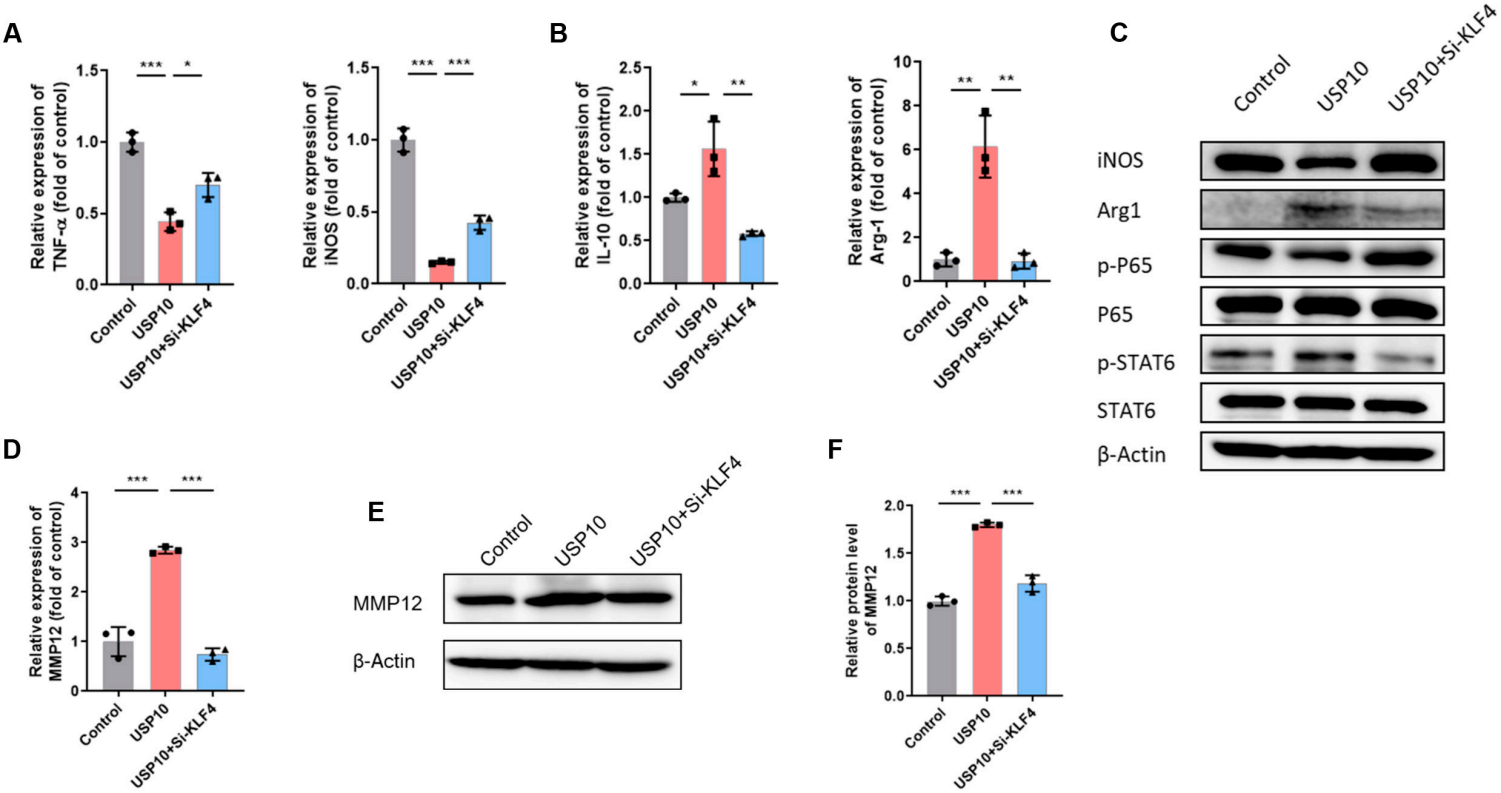
USP10 reprogrammed macrophage phenotype by stabilizing KLF4 expression. (A and B) qRT-PCR was used to determine the expression of M1 macrophage markers (TNF-α and iNOS) and M2 macrophage markers (IL-10 and Arg1) in LPS/IFN-γ-induced RAW264.7 cells transfected with empty vector control or USP10 expression plasmid, or with the combination treatment of USP10 expression plasmid + KLF4 siRNA. (C) Protein expression of iNOS, Arg1, p-P65, and p-STAT6 was analyzed in indicated groups. (D to F) MMP12 expression levels was measured in RAW264.7 cells transfected with control vector or USP10 expression plasmid, or with USP10 expression plasmid + KLF4 siRNA combination. Data are denoted as mean ± SD, *n* = 3; *P < 0.05, **P < 0.01, ***P < 0.001.

The above findings revealed that USP10 stabilizes KLF4 by inhibiting its ubiquitylation. Earlier research has demonstrated the significant function of KLF4 in macrophage polarization, inflammation, and scar repair [22,23]. We next inquired whether USP10 is involved in the regulation of macrophage phenotype by stabilizing KLF4 expression. Consistent with previous reports, our results firstly confirmed that KLF4 expression was down-regulated in pro-inflammatory macrophages, but significantly up-regulated in anti-inflammatory macrophages (Fig. S5A and B). Upon MSC-sEV treatment, the KLF4 protein level was obviously induced in pro-inflammatory macrophages (Fig. S5C and D). KLF4 overexpression in pro-inflammatory macrophages suppressed the expression of pro-inflammatory markers and the phosphorylation of P65. Meanwhile, it promoted the expression of anti-inflammatory markers and the phosphorylation level of STAT6 (Fig. S5E to G). To further explore the association between USP10 and KLF4 in regulating macrophage inflammation, USP10 was overexpressed with or without KLF4 knockdown in inflammatory macrophages. The findings demonstrated that KLF4 suppression markedly reversed the anti-inflammatory effect of USP10 overexpression on macrophages. This reversal was characterized by the up-regulation of M1 markers and the down-regulation of M2 markers. Moreover, the inhibition of pro-inflammatory signal and activation of anti-inflammatory signal induced by USP10 overexpression were abolished when KLF4 was silenced (Fig. 7A to C).

We also investigated whether USP10 modulates the expression of MMP12 via KLF4 in macrophages. The correlation between KLF4 and MMP12 in human normal liver tissues was firstly analyzed in the GEPIA database. As shown in Fig. S6A, there is an obvious positive correlation between them (*P* < 0.001). In in vitro experiments, we also confirmed that mRNA and protein levels of MMP12 were elevated by KLF4 overexpression, but suppressed by KLF4 knockdown (Fig. S6B to G). To further elucidate the specific mechanism of KLF4 regulating MMP12 expression, luciferase reporter assays were executed. According to the potential binding sites of MMP12 promoter regions, we constructed the corresponding binding site-directed mutation vectors. The luciferase assay findings indicated that the regulatory element appeared to be positioned within the region spanning from −1,633 to −1,157 base pairs (bp) (Fig. S6H). ChIP analysis further showed that KLF4 mainly binds to −1,310 and −1,078 bp in the MMP12 promoter (Fig. S6I to K). Next, to verify whether USP10 targeted KLF4 to regulate MMP12 expression, a rescue experiment was performed in vitro. As shown in Fig. 7D to F, USP10 overexpression resulted in an up-regulation of MMP12, while this effect was reversed by the silencing of KLF4.

### Engineered USP10-sEVs showed improved therapeutic efficacy in CCL4-induced liver fibrosis model

**Fig. 8.**
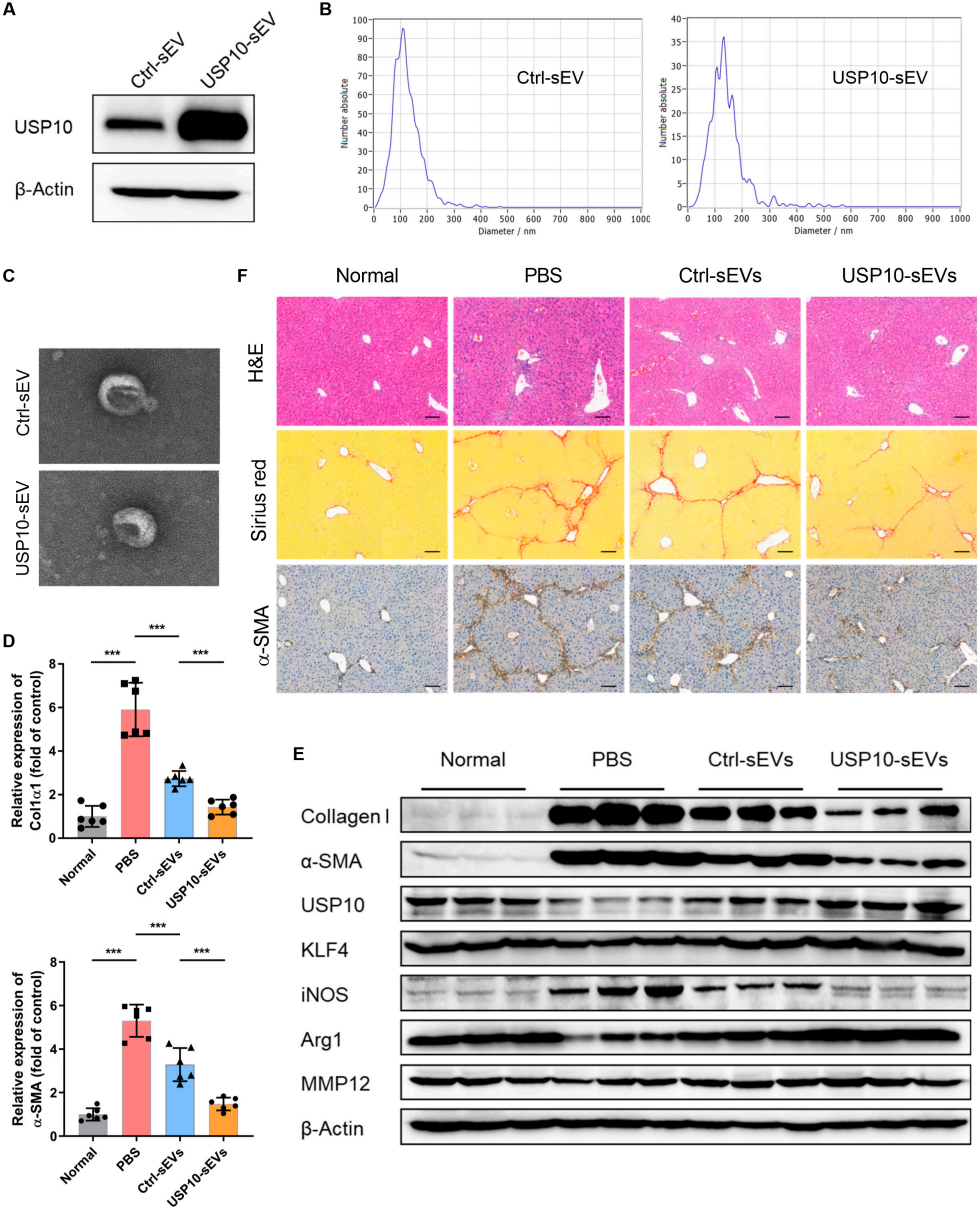
Therapeutic benefits of engineered USP10-sEVs in liver fibrosis. (A) USP10 level was examined by WB to demonstrate the overexpression efficiency. (B) Particle size distributions were assessed by NTA. (C) Morphology of engineered USP10-sEV was examined by TEM. (D) Relative mRNA expression of col1α1 and α-SMA in liver tissues was quantified by qRT-PCR. (E) WB with indicated antibodies to detect the protein levels in mouse liver tissues of different treatment groups. (F) Representative images of H&E, Sirius red, and α-SMA staining in liver tissues of different groups. Data are denoted as mean ± SD, *n* = 6 per group; ***P < 0.001.

Studies have demonstrated that genetic modifications of parent cells allow EVs to specifically enrich the desired contents [24,25]. Based on this, we generated USP10 stably overexpressing MSCs by lentiviral transfection and collected cell-cultured media to isolate USP10-overexpressed MSCs-sEVs, namely, USP10-sEV. Considering the potential influence of lentiviral transfection on MSCs-sEVs, we isolated sEVs from MSCs transfected with empty vector lentivirus as control group, namely, Ctrl-sEVs. Successful USP10 overexpression was confirmed by WB (Fig. 8A). The morphology of engineered USP10-sEVs remained intact through TEM and NTA analysis. Significant alteration in particle size was not observed (Fig. 8B and C). We also evaluated whether the genetic engineering process altered biodistribution or macrophage targeting efficiency. As shown in Fig. S7A and B, the fluorescence signals of administrated sEVs (labeled with DiR) were mainly accumulated in the liver, followed by the spleen and lung. No significant differences were observed between Ctrl-sEV and USP10-sEV groups for these parameters. To further track the cellular uptake of the engineered USP10-sEVs, the PKH67 dye was used as an indicator to label sEVs. The labeled Ctrl-sEVs or USP10-sEVs were administrated intravenously into fibrotic mice for 24 h. Liver NPCs were then separated and measured by flow cytometry. Consistent with previous studies [17,26], sEVs are predominantly taken up by macrophages. Flow cytometric results also revealed that USP10 engineering process did not affect the uptake of sEVs by hepatic macrophage. In in vitro experiments, PKH67-labeled Ctrl-sEVs or USP10-sEVs were cocultured with BMDMs for 12 h. The results also showed no difference in the proportion of PKH67^+^ cells or mean fluorescence intensity between 2 groups (Fig. S8A to C). All these results confirmed that the USP10 engineering process did not alter biodistribution or macrophage targeting efficiency of sEVs.

Subsequently, we assessed the therapeutic potential of USP10-sEVs in liver fibrosis mice. The experimental findings indicated that the efficacy of the USP10-sEV treatment group was superior to that of the Ctrl-sEV group. In the USP10-sEV treatment group, the expression of collagen I and α-SMA was reduced at both mRNA and protein levels versus the Ctrl-sEV group, with significant statistical differences. Additionally, USP10 and KLF4 levels in the livers of mice treated with USP10-sEVs were significantly up-regulated compared to those in both PBS and Ctrl-sEV groups. Following USP10-sEV treatment, there was a significant down-regulation of pro-inflammatory marker iNOS, whereas anti-inflammatory marker Arg1 and matrix metalloproteinase MMP12 expression were notably up-regulated (Fig. 8D and E). Consistent with these results, histopathological staining also suggested more pronounced improvements in inflammatory cell infiltration and tissue structure in the USP10-sEV treatment group, as well as further reductions in collagen deposition and α-SMA staining area compared to the Ctrl-sEV group (Fig. 8F). Collectively, these data demonstrate that engineered USP10-sEVs efficiently ameliorated liver fibrosis in vivo.

## Discussion

Liver fibrosis is a common consequence of chronic liver injury, which can lead to irreversible cirrhosis, liver cancer, and eventually end-stage liver failure. Liver transplantation remains the only effective treatment for individuals with end-stage liver disease. Nevertheless, the critical scarcity of available liver donors restricts its broad implementation. MSC is a kind of adult stem cell extensively employed in preclinical and clinical investigations of liver fibrosis/cirrhosis, demonstrating promising prospects [27]. However, the precise therapeutic mechanism remains to be elucidated. Beyond their ability to differentiate into tissue-specific cells, MSCs also release a wide range of factors such as cytokines, chemokines, and growth factors. Thus, they have also been called the “drug store” [28]. In our previous study, we have reported that MSCs exert anti-fibrotic and anti-inflammatory effects through TSG-6 in a paracrine manner [29]. In the current study, we observed that MSC transplantation could effectively ameliorate liver fibrosis in CCL4-induced mouse model, primarily through sEV-mediated communication with macrophages. USP10 was identified as the key effector responsible for the anti-fibrotic effects exerted by MSCs-sEVs. Moreover, a new mechanism is elucidated, demonstrating that USP10 deubiquitinates and stabilizes KLF4, thereby regulating the NF-κB/STAT6 signaling and the transcription of MMP12.

Recently, it has been recognized that sEVs are important bioactive mediators, with increasing expectations for their potential as a cell-free therapeutic approach. MSCs-sEVs naturally mirror their parental cell properties and replicate the beneficial effects of MSCs in liver injury. Growing evidence supports that MSCs-sEVs inhibit cell death and oxidative stress, support hepatocyte function, and exert immunomodulatory effects for liver disease [9]. In our study, we found that both MSCs and MSCs-sEVs exhibited therapeutic effects on CCL4-induced liver fibrosis in mice. A significant observation revealed that the effectiveness of MSCs was markedly diminished when a vesicle release inhibitor (GW4869) was utilized, highlighting the critical role of sEV in MSCs’ anti-fibrotic effects. More importantly, in contrast to MSC therapy, sEVs have the benefits of greater safety, easier preservation, fewer side effects, and fewer ethical dilemmas. Therefore, MSCs-sEVs may achieve a better clinical application.

Although the activation of HSCs represents a crucial process in both initiating and advancing liver fibrosis, the function of immune cells, specifically macrophages, exhibits substantial influence over HSC activation regulation [30]. Depleting macrophages in mice during the progressive phase of liver fibrosis decreased collagen formation, while their removal during the regression phase impeded liver tissue repair. Our study found that depleting liver macrophages with chlorophosphate liposomes negated the anti-fibrotic effects of MSCs-sEVs. These results highlight the crucial role of macrophages in both liver injury and repair processes, emphasizing their potential as a therapeutic target for treating liver fibrosis. Currently, the classical therapeutic strategies targeting liver fibrosis in macrophages involve manipulating their polarization, reprogramming and enhancing autophagy, as well as reducing the recruitment of infiltrating macrophages [10]. Our data confirmed that MSCs-sEVs suppressed inflammatory responses in macrophages, and thus modulated the immune microenvironment of the liver. In addition, macrophages are an important source of MMP production [31]. We also found that MSCs-sEVs up-regulates MMP12 expression that contributes to tissue remodeling in liver fibrosis.

Cargo molecules, such as microRNAs or proteins, could be carried by sEVs for intercellular communication and material transfer. Our previous study demonstrated that MSCs-derived exosomes exert protective effects against liver fibrosis by delivering miR-148a to macrophages, which targets the KLF6/STAT3 pathway [18]. Similarly, some noncoding RNA targets including miR-27b-3p [16], miR-124 [32], and circDIDO1 [33] are identified in MSCs-sEVs, which are viable for attenuating liver fibrosis. In addition to transcription regulation, sEVs also play crucial roles in the posttranslational regulation of recipient cells. Chamberlain et al. [34] demonstrated that SIRT2 was transferred from oligodendrocytes to axonal through exosome, enhancing energy metabolism by deacetylating mitochondrial proteins. In addition to acetylation, exosome-mediated ubiquitination modification has also recently been reported [35]. Interestingly, our proteomic analysis also identified many ubiquitination-related molecules in MSCs-sEVs. Prior investigations have demonstrated that ubiquitination serves a pivotal function in regulating the signaling pathways of innate immunity and modulating the production of inflammatory factors [21]. Liu et al. [36] reported that USP19 inhibits inflammation and enhances M2-like macrophage polarization by modulating the function of NLRP3. Additionally, studies have shown that USP29 could participate in the repair of spinal cord injury by regulating microglia polarization [37]. In our study, we found that USP10 was most significantly enriched in MSCs-sEVs. Research on USP10’s role in liver fibrosis and cirrhosis is limited. Herein, we noted that the USP10 level was notably diminished in cirrhotic liver. The therapeutic effect of MSCs-sEVs in remodeling macrophage phenotype and alleviating liver fibrosis is diminished when USP10 is knocked down, highlighting its significance.

Further investigation revealed the potential mechanism underlying the anti-fibrotic effects of MSCs-sEVs, with KLF4 identified as a key interaction protein for USP10 in macrophages. KLF4 has been recognized as a crucial factor in macrophage regulation, functioning through the NF-kB and STAT6 pathways [22]. Research has demonstrated that posttranslational modifications such as acetylation, phosphorylation, and ubiquitination can regulate KLF4 expression and activity [38,39]. Our findings demonstrate that USP10 interacts and promotes deubiquitination and stabilization of KLF4 in macrophages. Inhibition of KLF4 reversed the impact of USP10 overexpression on macrophage polarization, showing that KLF4 is important for licensing the anti-inflammatory properties of macrophages. Moreover, we have shown for the first time that KLF4 participates in the transcriptional regulation of MMP12 in macrophages. Notably, MMP13, another key enzyme involved in matrix remodeling during the regression phase of liver fibrosis, was also analyzed in our study. However, we did not detect significant changes. We analyzed the potential KLF4-binding sites on MMP12 and MMP13 gene promoter by using JASPAR database (https://jaspar.elixir.no/). More KLF4-binding sites and higher binding scores are detected in MMP12 gene promoter region. Correlation analysis in the GEPIA database further confirmed that KLF4 showed significant correlation with MMP12 (Fig. S6A), but not with MMP13 (Fig. S9). Thus, MMP12 was identified as the main effector mediating extracellular matrix (ECM) remodeling in MSCs-sEV-treated macrophages. The USP10–KLF4–MMP12 axis deepens our understanding of how MSCs-sEVs achieve anti-fibrotic effects, also providing a theoretical foundation for subsequent engineered MSCs-sEVs.

Recent studies have developed a gene therapy approach utilizing dendrimer–graphite nanoparticles to selectively enhance RNF41 expression in macrophages, leading to notable improvements in hepatic fibrosis and regeneration in liver injury mouse models [40]. As natural nanomaterials, EVs have been proposed as promising liver-accumulating drug delivery vehicles for the treatment of liver diseases [17]. So, MSCs-sEVs were also utilized as therapeutic vehicles in our study to develop engineered sEVs. The results of in vivo experiments further confirmed that engineered USP10-sEV had superior functions in inhibiting the inflammatory response and promoting the degradation of ECM, thus achieving the optimized antifibrotic efficacy.

Alongside the insights provided by our findings, we acknowledge the presence of limitations in relation to this study. Firstly, while hepatic macrophages are primarily responsible for the uptake of circulating sEVs, it is important to consider other cells within the liver microenvironment, including HSCs, B cells, and liver sinusoidal endothelial cells (LSECs). For example, Liu et al. [41] reported that MSCs-EVs loaded with miR-181a-5p attenuated HSC activation by inhibiting the transforming growth factor β (TGFβ)/Smad signaling pathways. More importantly, the authors augmented therapeutic efficacy by engineering sEVs with surface modifications to overcome poor targeting of HSCs. Similarly, peptide-mediated surface functionalization endows exosomes with good LSEC-targeting ability for the treatment of liver injuries [42]. In a recent study, Mao et al. [43] confirmed that USP10 overexpression could alleviate fibroblast activation leading to an attenuation of lung fibrosis. So, it would be interesting to use surface modifications to enhance the targeting of HSCs to further evaluate the therapeutic effects of USP10-sEVs for liver fibrosis. Secondly, MSCs-sEVs possess various bioactive molecules that contribute to their positive impact on fibrosis. Other undisclosed molecular mechanisms deserve further study. Lastly, numerous challenges persist in the clinical application of MSCs-sEVs including large-scale production of sEVs, quality control measures, and implementation strategies. These aspects remain open for continued exploration and standardization.

**Fig. 9.**
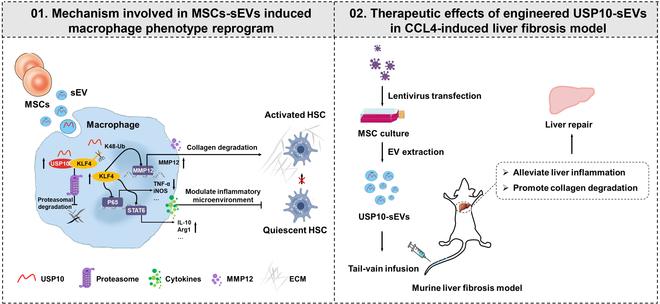
Schematic overview of the study. MSCs-sEVs protected against liver fibrosis by delivering USP10 that reprogrammed macrophage phenotypes to promote their anti-inflammatory and repairing properties. Mechanistically, USP10 stabilizes the expression of KLF4 through deubiquitination, thereby regulating NF-κB/STAT6 signaling pathways and participating in the transcriptional regulation of MMP12. Moreover, USP10-enriched engineered MSCs-sEVs further potentiated their antifibrotic effects.

In conclusion, MSCs-sEVs effectively ameliorated liver fibrosis by inducing phenotypic transformation of macrophages to promote their anti-inflammatory and reparative properties. The deubiquitinase USP10 has been identified as a crucial effector molecule in this process. Mechanistically, USP10 deubiquitinates and stabilizes KLF4 in macrophages, thereby regulating the NF-κB/STAT6 signaling pathways and participating in the transcriptional regulation of MMP12. Additionally, we have successfully developed USP10-modified engineered sEVs based on these findings, which further augment the therapeutic effectiveness of MSCs-sEVs (Fig. 9).

## Data Availability

The data that support the findings can be obtained from the corresponding author on reasonable request.

## References

[1] Asrani SK, Devarbhavi H, Eaton J, Kamath PS. Burden of liver diseases in the world. J Hepatol. 2019;70(1):151–171. 30266282 10.1016/j.jhep.2018.09.014

[2] Bataller R, Brenner DA. Liver fibrosis. J Clin Invest. 2005;115(2):209–218. 15690074 10.1172/JCI24282 PMC546435

[3] Zanetto A, Northup P, Roberts L, Senzolo M. Haemostasis in cirrhosis: Understanding destabilising factors during acute decompensation. J Hepatol. 2023;78(5):1037–1047. 36708812 10.1016/j.jhep.2023.01.010

[4] Yao L, Hu X, Dai K, Yuan M, Liu P, Zhang Q, Jiang Y. Mesenchymal stromal cells: Promising treatment for liver cirrhosis. Stem Cell Res Ther. 2022;13(1): Article 308. 35841079 10.1186/s13287-022-03001-z PMC9284869

[5] Shi M, Li YY, Xu RN, Meng FP, Yu SJ, Fu JL, Hu JH, Li JX, Wang LF, Jin L, et al. Mesenchymal stem cell therapy in decompensated liver cirrhosis: A long-term follow-up analysis of the randomized controlled clinical trial. Hepatol Int. 2021;15(6):1431–1441. 34843069 10.1007/s12072-021-10199-2 PMC8651584

[6] Liu Y, Dong Y, Wu X, Xu X, Niu J. The assessment of mesenchymal stem cells therapy in acute on chronic liver failure and chronic liver disease: A systematic review and meta-analysis of randomized controlled clinical trials. Stem Cell Res Ther. 2022;13(1): Article 204. 35578365 10.1186/s13287-022-02882-4 PMC9109309

[7] Alfaifi M, Eom YW, Newsome PN, Baik SK. Mesenchymal stromal cell therapy for liver diseases. J Hepatol. 2018;68(6):1272–1285. 29425678 10.1016/j.jhep.2018.01.030

[8] Levy O, Kuai R, Siren EMJ, Bhere D, Milton Y, Nissar N, De Biasio M, Heinelt M, Reeve B, Abdi R, et al. Shattering barriers toward clinically meaningful MSC therapies. Sci Adv. 2020;6(30): Article eaba6884. 32832666 10.1126/sciadv.aba6884 PMC7439491

[9] Psaraki A, Ntari L, Karakostas C, Korrou-Karava D, Roubelakis MG. Extracellular vesicles derived from mesenchymal stem/stromal cells: The regenerative impact in liver diseases. Hepatology. 2022;75(6):1590–1603. 34449901 10.1002/hep.32129

[10] Cheng D, Chai J, Wang H, Fu L, Peng S, Ni X. Hepatic macrophages: Key players in the development and progression of liver fibrosis. Liver Int. 2021;41(10):2279–2294. 33966318 10.1111/liv.14940

[11] Wynn TA, Vannella KM. Macrophages in tissue repair, regeneration, and fibrosis. Immunity. 2016;44(3):450–462. 26982353 10.1016/j.immuni.2016.02.015 PMC4794754

[12] Kisseleva T, Brenner D. Molecular and cellular mechanisms of liver fibrosis and its regression. Nat Rev Gastroenterol Hepatol. 2021;18(3):151–166. 33128017 10.1038/s41575-020-00372-7

[13] Ramachandran P, Iredale JP. Macrophages: Central regulators of hepatic fibrogenesis and fibrosis resolution. J Hepatol. 2012;56(6):1417–1419. 22314426 10.1016/j.jhep.2011.10.026

[14] Mardpour S, Ghanian MH, Sadeghi-Abandansari H, Mardpour S, Nazari A, Shekari F, Baharvand H. Hydrogel-mediated sustained systemic delivery of mesenchymal stem cell-derived extracellular vesicles improves hepatic regeneration in chronic liver failure. ACS Appl Mater Interfaces. 2019;11(41):37421–37433. 31525863 10.1021/acsami.9b10126

[15] Yuan M, Yao L, Chen P, Wang Z, Liu P, Xiong Z, Hu X, Li L, Jiang Y. Human umbilical cord mesenchymal stem cells inhibit liver fibrosis via the microRNA-148a-5p/SLIT3 axis. Int Immunopharmacol. 2023;125(Pt A): Article 111134. 37918086 10.1016/j.intimp.2023.111134

[16] Cheng F, Yang F, Wang Y, Zhou J, Qian H, Yan Y. Mesenchymal stem cell-derived exosomal miR-27b-3p alleviates liver fibrosis via downregulating YAP/LOXL2 pathway. J Nanobiotechnol. 2023;21(1): Article 195. 10.1186/s12951-023-01942-y PMC10273609 37328872

[17] Zhang G, Huang X, Xiu H, Sun Y, Chen J, Cheng G, Song Z, Peng Y, Shen Y, Wang J, et al. Extracellular vesicles: Natural liver-accumulating drug delivery vehicles for the treatment of liver diseases. J Extracell Vesicles. 2020;10(2): Article e12030. 33335695 10.1002/jev2.12030 PMC7726052

[18] Tian S, Zhou X, Zhang M, Cui L, Li B, Liu Y, Su R, Sun K, Hu Y, Yang F, et al. Mesenchymal stem cell-derived exosomes protect against liver fibrosis via delivering miR-148a to target KLF6/STAT3 pathway in macrophages. Stem Cell Res Ther. 2022;13(1): Article 330. 35858897 10.1186/s13287-022-03010-y PMC9297598

[19] Zhang M, Tian SY, Ma SY, Zhou X, Zheng XH, Li B, Guo GY, Yu JH, Su R, Yang FF, et al. Deficient chaperone-mediated autophagy in macrophage aggravates inflammation of nonalcoholic steatohepatitis by targeting Nup85. Liver Int. 2023;43(5):1021–1034. 36912786 10.1111/liv.15547

[20] Zhou J, Yang J, Fan X, Hu S, Zhou F, Dong J, Zhang S, Shang Y, Jiang X, Guo H, et al. Chaperone-mediated autophagy regulates proliferation by targeting RND3 in gastric cancer. Autophagy. 2016;12(3):515–528. 26761524 10.1080/15548627.2015.1136770 PMC4836009

[21] Lin Z, Yang P, Hu Y, Xu H, Duan J, He F, Dou K, Wang L. RING finger protein 13 protects against nonalcoholic steatohepatitis by targeting STING-relayed signaling pathways. Nat Commun. 2023;14(1): Article 6635. 37857628 10.1038/s41467-023-42420-1 PMC10587083

[22] Liao X, Sharma N, Kapadia F, Zhou G, Lu Y, Hong H, Paruchuri K, Mahabeleshwar GH, Dalmas E, Venteclef N, et al. Krüppel-like factor 4 regulates macrophage polarization. J Clin Invest. 2011;121(7):2736–2749. 21670502 10.1172/JCI45444 PMC3223832

[23] Wang J, Zhao M, Zhang H, Yang F, Luo L, Shen K, Wang X, Li Y, Zhang J, Zhang J, et al. KLF4 alleviates hypertrophic scar fibrosis by directly activating BMP4 transcription. Int J Biol Sci. 2022;18(8):3324–3336. 35637963 10.7150/ijbs.71167 PMC9134901

[24] Hu C, Zhao L, Li L. Genetic modification by overexpression of target gene in mesenchymal stromal cell for treating liver diseases. J Mol Med. 2021;99(2):179–192. 33388882 10.1007/s00109-020-02031-5

[25] Zhu W, Wang Q, Zhang J, Sun L, Hong X, Du W, Duan R, Jiang J, Ji Y, Wang H, et al. Exosomes derived from mir-214-3p overexpressing mesenchymal stem cells promote myocardial repair. Biomater Res. 2023;27(1): Article 77. 37563655 10.1186/s40824-023-00410-w PMC10413540

[26] Imai T, Takahashi Y, Nishikawa M, Kato K, Morishita M, Yamashita T, Matsumoto A, Charoenviriyakul C, Takakura Y. Macrophage-dependent clearance of systemically administered B16BL6-derived exosomes from the blood circulation in mice. J Extracell Vesicles. 2015;4: Article 26238. 25669322 10.3402/jev.v4.26238 PMC4323410

[27] Dwyer BJ, Macmillan MT, Brennan PN, Forbes SJ. Cell therapy for advanced liver diseases: Repair or rebuild. J Hepatol. 2021;74(1):185–199. 32976865 10.1016/j.jhep.2020.09.014

[28] Caplan AI, Correa D. The MSC: An injury drugstore. Cell Stem Cell. 2011;9(1):11–15. 21726829 10.1016/j.stem.2011.06.008 PMC3144500

[29] Wang M, Zhang M, Fu L, Lin J, Zhou X, Zhou P, Huang P, Hu H, Han Y. Liver-targeted delivery of TSG-6 by calcium phosphate nanoparticles for the management of liver fibrosis. Theranostics. 2020;10(1):36–49. 31903104 10.7150/thno.37301 PMC6929629

[30] Duffield JS, Forbes SJ, Constandinou CM, Clay S, Partolina M, Vuthoori S, Wu S, Lang R, Iredale JP. Selective depletion of macrophages reveals distinct, opposing roles during liver injury and repair. J Clin Invest. 2005;115(1):56–65. 15630444 10.1172/JCI22675 PMC539199

[31] Zhao X, Chen J, Sun H, Zhang Y, Zou D. New insights into fibrosis from the ECM degradation perspective: The macrophage-MMP-ECM interaction. Cell Biosci. 2022;12(1): Article 117. 35897082 10.1186/s13578-022-00856-w PMC9327238

[32] Niknam B, Baghaei K, Mahmoud Hashemi S, Hatami B, Reza Zali M, Amani D. Human Wharton’s jelly mesenchymal stem cells derived-exosomes enriched by miR-124 promote an anti-fibrotic response in an experimental model of liver fibrosis. Int Immunopharmacol. 2023;119: Article 110294. 37167639 10.1016/j.intimp.2023.110294

[33] Ma L, Wei J, Zeng Y, Liu J, Xiao E, Kang Y, Kang Y. Mesenchymal stem cell-originated exosomal circDIDO1 suppresses hepatic stellate cell activation by miR-141-3p/PTEN/AKT pathway in human liver fibrosis. Drug Deliv. 2022;29(1):440–453. 35099348 10.1080/10717544.2022.2030428 PMC8812765

[34] Chamberlain KA, Huang N, Xie Y, LiCausi F, Li S, Li Y, Sheng ZH. Oligodendrocytes enhance axonal energy metabolism by deacetylation of mitochondrial proteins through transcellular delivery of SIRT2. Neuron. 2021;109(21):3456–3472.e8. 34506725 10.1016/j.neuron.2021.08.011 PMC8571020

[35] Liang M, Chen X, Wang L, Qin L, Wang H, Sun Z, Zhao W, Geng B. Cancer-derived exosomal TRIM59 regulates macrophage NLRP3 inflammasome activation to promote lung cancer progression. J Exp Clin Cancer Res. 2020;39(1): Article 176. 32867817 10.1186/s13046-020-01688-7 PMC7457778

[36] Liu T, Wang L, Liang P, Wang X, Liu Y, Cai J, She Y, Wang D, Wang Z, Guo Z, et al. USP19 suppresses inflammation and promotes M2-like macrophage polarization by manipulating NLRP3 function via autophagy. Cell Mol Immunol. 2021;18(10):2431–2442. 33097834 10.1038/s41423-020-00567-7 PMC8484569

[37] Liu W, Tang P, Wang J, Ye W, Ge X, Rong Y, Ji C, Wang Z, Bai J, Fan J, et al. Extracellular vesicles derived from melatonin-preconditioned mesenchymal stem cells containing USP29 repair traumatic spinal cord injury by stabilizing NRF2. J Pineal Res. 2021;71(4): Article e12769. 34562326 10.1111/jpi.12769

[38] Jha K, Kumar A, Bhatnagar K, Patra A, Bhavesh NS, Singh B, Chaudhary S. Modulation of Krüppel-like factors (KLFs) interaction with their binding partners in cancers through acetylation and phosphorylation. Biochim Biophys Acta Gene Regul Mech. 2024;1867(1): Article 195003. 37992989 10.1016/j.bbagrm.2023.195003

[39] Wang X, Xia S, Li H, Wang X, Li C, Chao Y, Zhang L, Han C. The deubiquitinase USP10 regulates KLF4 stability and suppresses lung tumorigenesis. Cell Death Differ. 2020;27(6):1747–1764. 31748695 10.1038/s41418-019-0458-7 PMC7244734

[40] Moreno-Lanceta A, Medrano-Bosch M, Fundora Y, Perramón M, Aspas J, Parra-Robert M, Baena S, Fondevila C, Edelman ER, Jiménez W, et al. RNF41 orchestrates macrophage-driven fibrosis resolution and hepatic regeneration. Sci Transl Med. 2023;15(704): Article eabq6225. 37437019 10.1126/scitranslmed.abq6225 PMC10712730

[41] Liu Y, Chen S, Huang H, Midgley AC, Han Z, Han ZC, Li Q, Li Z. Ligand-tethered extracellular vesicles mediated RNA therapy for liver fibrosis. Adv Healthc Mater. 2025;14(2): Article e2403068. 39520385 10.1002/adhm.202403068

[42] Du W, Chen C, Liu Y, Quan H, Xu M, Liu J, Song P, Fang Z, Yue Z, Xu H, et al. A combined “eat me/don’t eat me” strategy based on exosome for acute liver injury treatment. Cell Rep Med. 2025;6(4): Article 102033. 40120577 10.1016/j.xcrm.2025.102033 PMC12047510

[43] Mao S, Yu N, Wang W, Mao Y, Du Y, Zhao Q, Gu X, Kang J. Ubiquitin-specific peptidase 10 attenuates bleomycin-induced pulmonary fibrosis via modulating autophagy depending on sirtuin 6-mediated AKT/mTOR. Cell Biol Toxicol. 2025;41(1): Article 73. 40278953 10.1007/s10565-025-10031-9 PMC12031808

