# Supplementary material for: Engineered Mesenchymal Stem Cell-Derived Small Extracellular Vesicles Mitigate Liver Fibrosis by Delivering USP10 to Reprogram Macrophage Phenotype
Source: Biomater Res. 2025 Aug 26;29:0244. doi: 10.34133/bmr.0244 (PMC12380376; doi:10.34133/bmr.0244)
Supplement: Supplementary 1 — Figs. S1 to S9 Tables S1 and S2 [file bmr.0244.f1.docx]

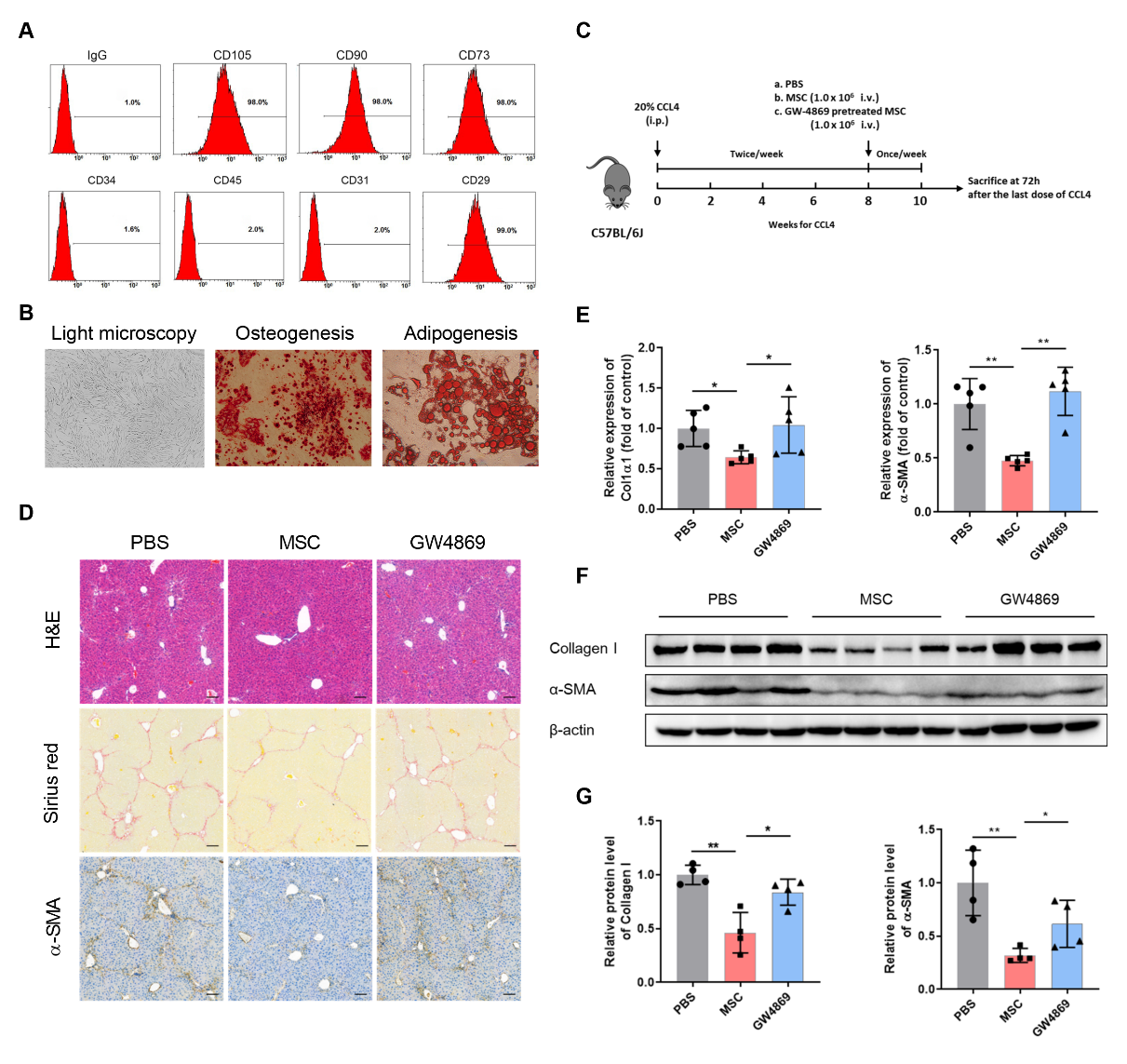


**Supplementary Figure 1. GW4869 pre-treatment impaired therapeutic effects of MSCs in ameliorating CCL4-induced liver fibrosis.** A) Characterization of surface markers by flow cytometry. B) Morphological features of MSCs under different culture conditions. C) Schematic illustration of grouping and different treatments applied. D) Representative images of HE, Sirius red and α-SMA staining in liver tissues of different groups. Scale bar 100 μm. E) Relative mRNA expression levels of col1α1 and α-SMA in liver tissues of diverse groups. F-G) WB analysis of protein levels of collagen I and α-SMA in liver tissues of different groups. Data are presented as mean ± SD, n=4-5 per group. *p < 0.05, **p < 0.01.


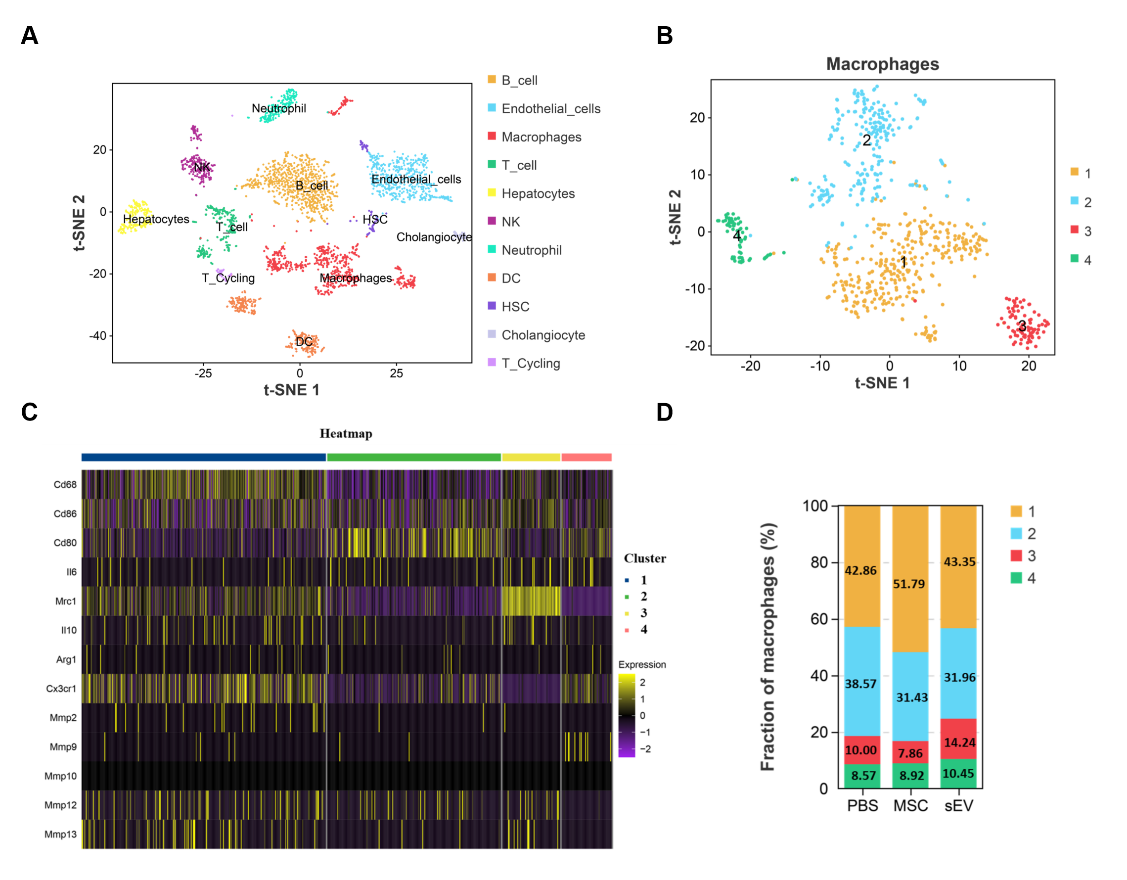


**Supplementary Figure 2. Single-cell transcriptome analysis of liver samples.** A) T-SNE plot of 11 cell clusters in combined analysis of PBS-, MSC- or sEV-treated group. B) T-SNE plot showing 4 subpopulations of macrophages. C) Expression profiles of classical pro- and anti-inflammatory marker as well as matrix metalloproteins in macrophage subpopulations. D) The proportion of 4 macrophage clusters in different treatment groups.

**
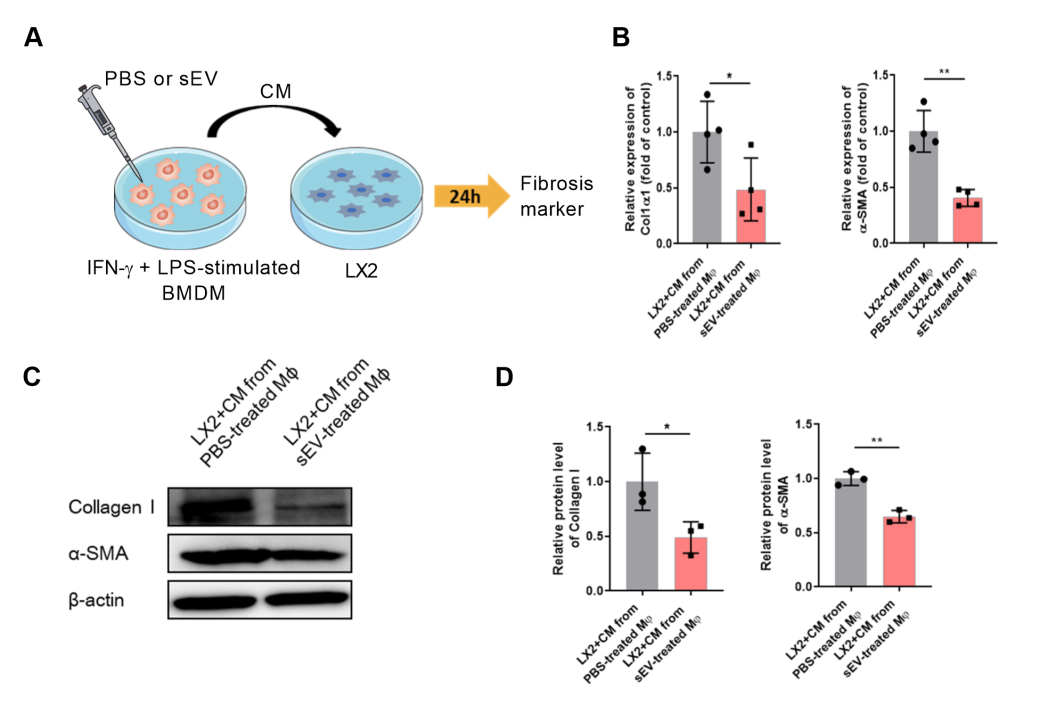
**

**Supplementary Figure 3. MSCs-sEVs suppressed macrophage-mediated** **hepatic stellate cell activation under inflammatory conditions.** A) Schematic diagram of LX2 co-cultured with BMDMs supernatant. B) Quantitative real-time PCR analysis for the transcriptional expression levels of fibrosis markers. C-D) Protein expression of collagen I and α-SMA was analysed by western-blot. Data are presented as mean ± SD, n=3; *p < 0.05, **p < 0.01.


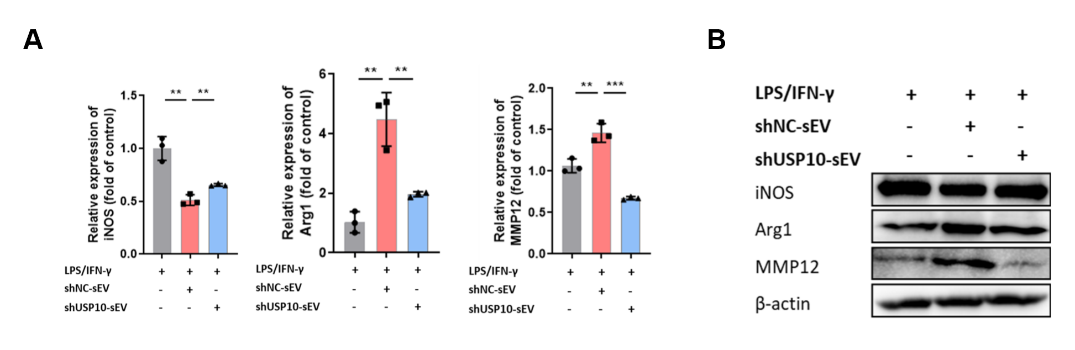


**Supplementary Figure 4. Silencing USP10 in MSCs-sEVs impaired the ability of MSCs-sEVs to suppress inflammation and promote MMP12 expression in BMDMs.** A) Quantitative real-time PCR analysis for the transcriptional expression levels of iNOS, Arg1 and MMP12 in the different treated groups. B) Protein expression of iNOS, Arg1 and MMP12 was analysed by western-blot in the different treated groups. Data are presented as mean ± SD, n=3; **p < 0.01, ***p < 0.001.


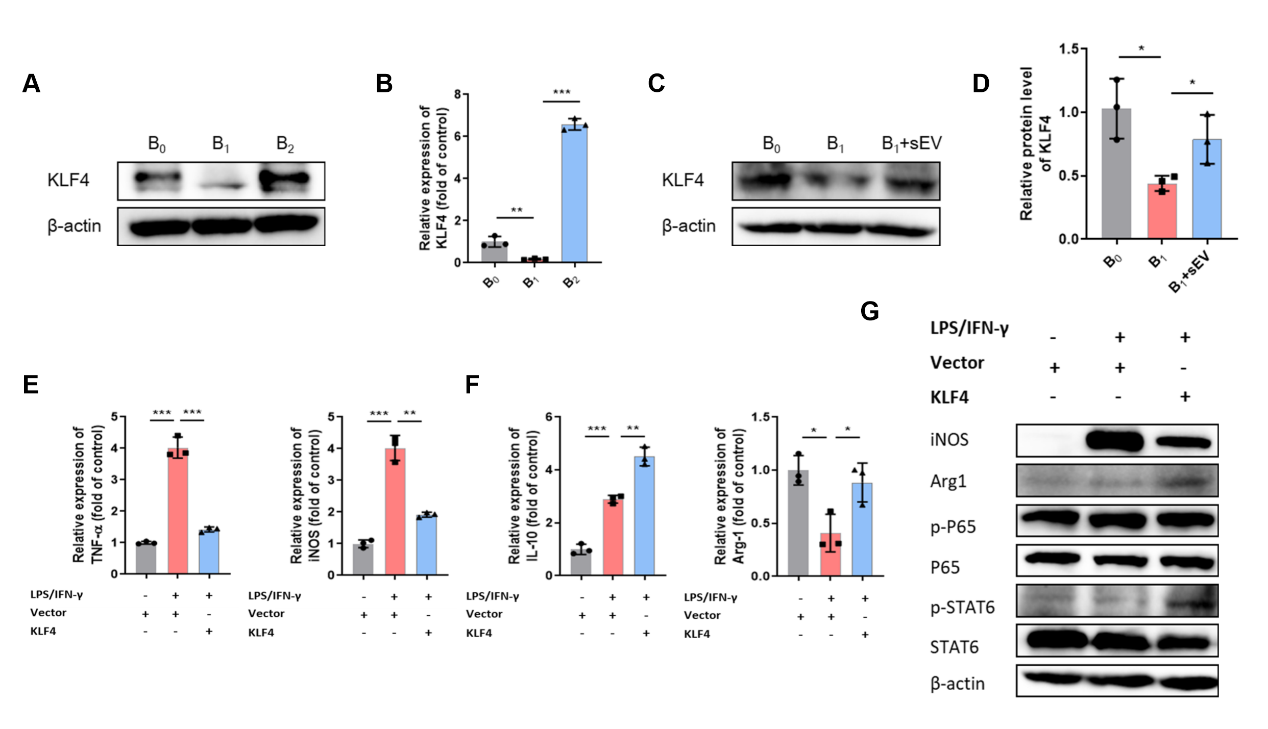


**Supplementary Figure 5. KLF4 regulated M1/M2 macrophage polarization by P65 and STAT6 pathways.** A-B) KLF4 level was measured in different phenotypes of BMDMs by WB and qRT-PCR. C-D) The KLF4 protein level in BMDMs was assessed by WB analysis after LPS/IFN-γ stimulation with or without MSCs-sEVs treatment. E-F) qRT-PCR was applied to determine M1 macrophage markers (TNF-α and iNOS) and M2 macrophage markers (IL-10 and Arg1) levels in RAW264.7 cells transfected with empty vector control or KLF4 expression plasmid in different stimulation conditions. G) iNOS, Arg1, p-P65 and p-STAT6 level was analysed in indicated groups. Data are presented as mean ± SD, n=3; *p < 0.05, **p < 0.01, ***p < 0.001.


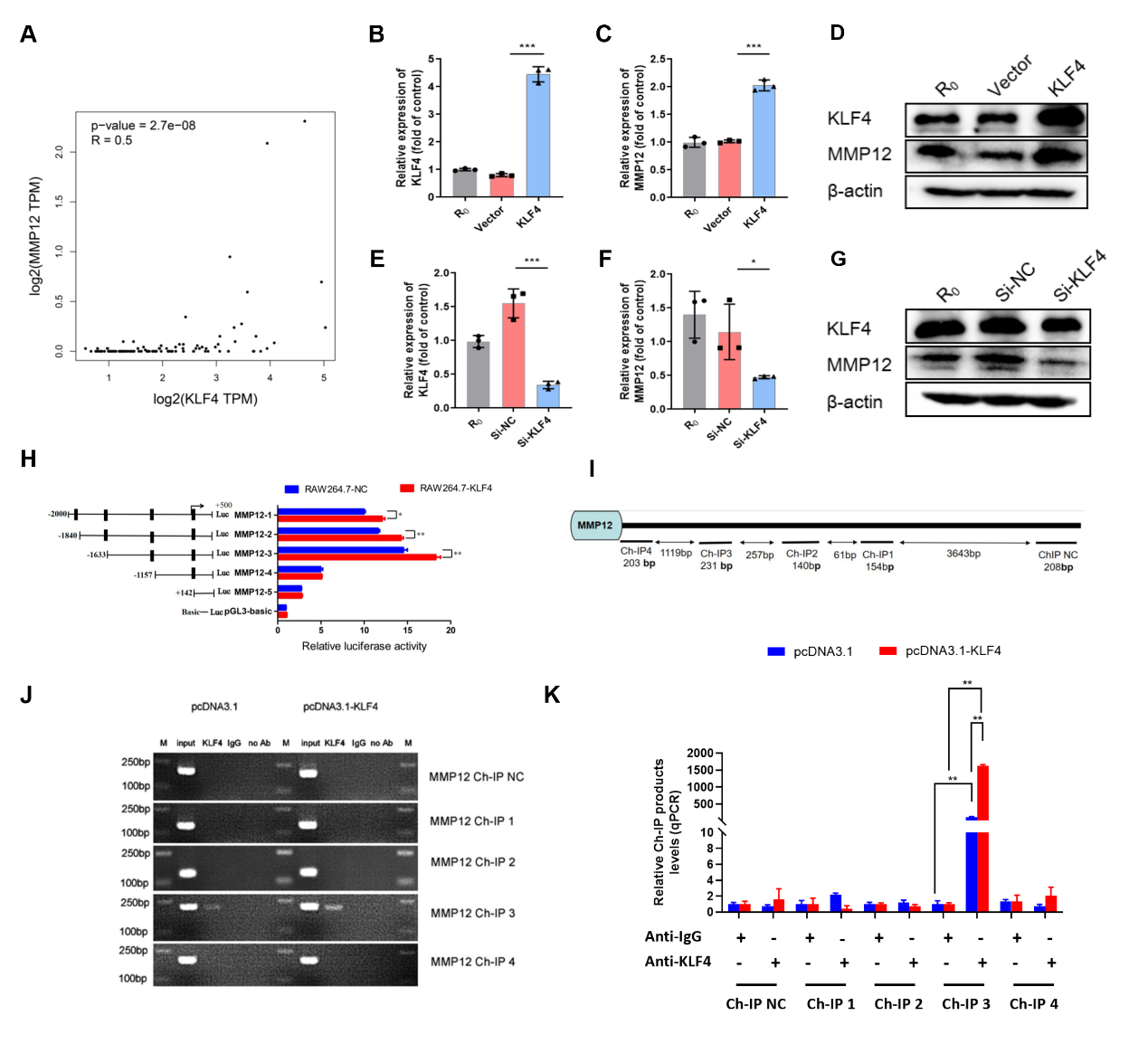


**Supplementary Figure 6. KLF4 promoted MMP12 expression of macrophages at the transcriptional level.** A) A notable positive link between KLF4 and MMP12 expression was noted in human normal liver tissues. B) Overexpression efficiency of KLF4 in RAW264.7 cells was validated utilizing qRT-PCR. C-D) The mRNA and protein level of MMP12 was measured using qRT-PCR and WB. E) KLF4 silencing efficiency in RAW264.7 cells was validated through qRT-PCR. F-G) MMP12 expression levels, both mRNA and protein, underwent analysis via qRT-PCR and WB. H) Luciferase reporter analysis of RAW264.7 cells following transfection with pcDNA-KLF4 and sequential MMP12 promoter luciferase constructs. I) JASPAR database facilitated the identification of potential KLF4 binding regions on the MMP12 promoter. J-K) ChIP-qPCR analysis demonstrated KLF4’s direct interaction with the MMP12 promoter. Data are presented as mean ± SD, n=3; ***p < 0.001.


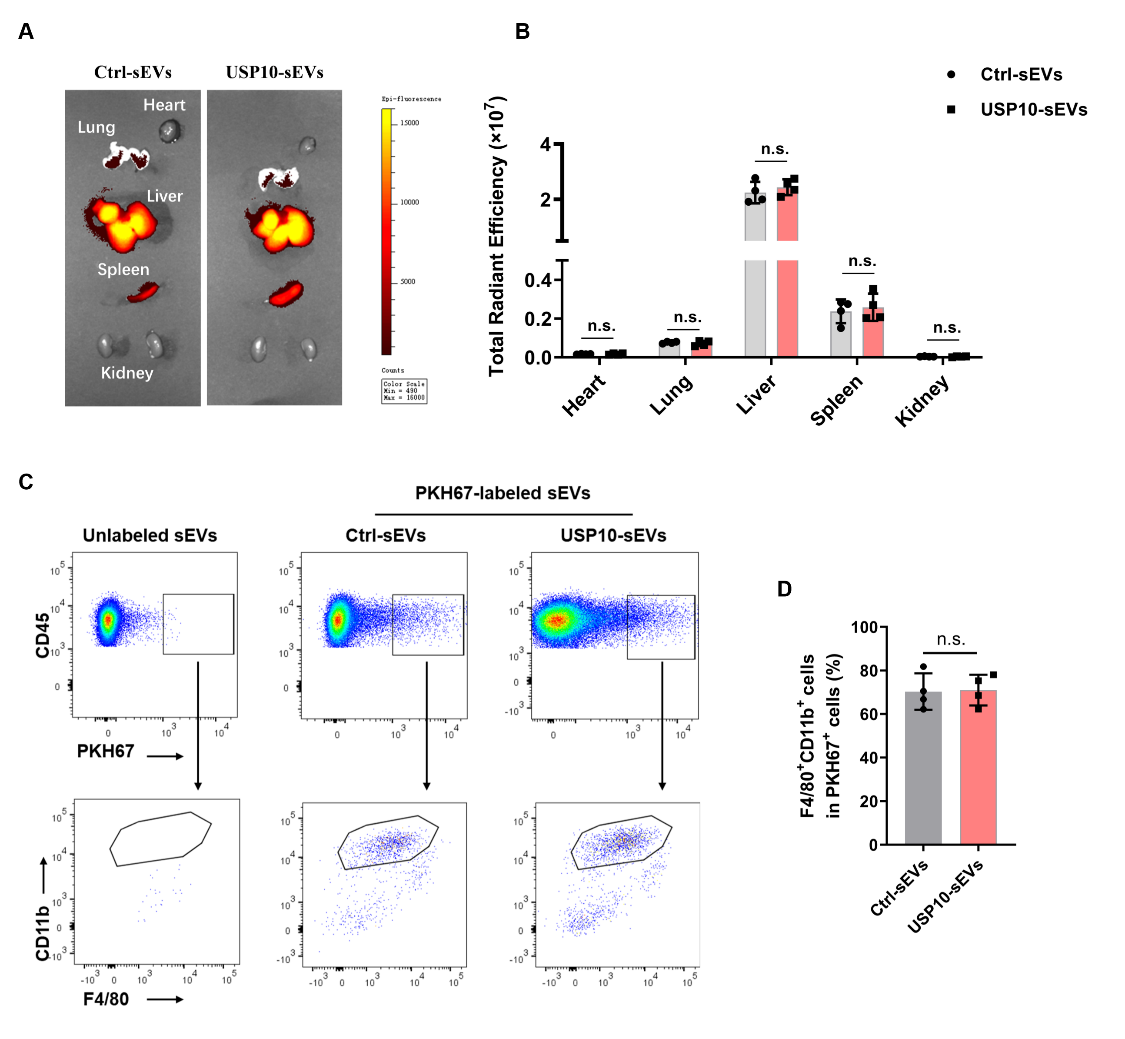


**Supplementary Figure 7. Biodistribution of the engineered USP10-sEVs in CCL4-induced liver fibrosis model.** A-B) DiR signals in the different organs were examined by using an in vivo imaging system. C-D) Uptake of PKH67-labeled Ctrl-sEVs or USP10-sEVs by F4/80^+^CD11b^+^ macrophages in the liver was detected by flow cytometry plots. Data are denoted as mean ± SD, n=4 per group; n.s. no significance.


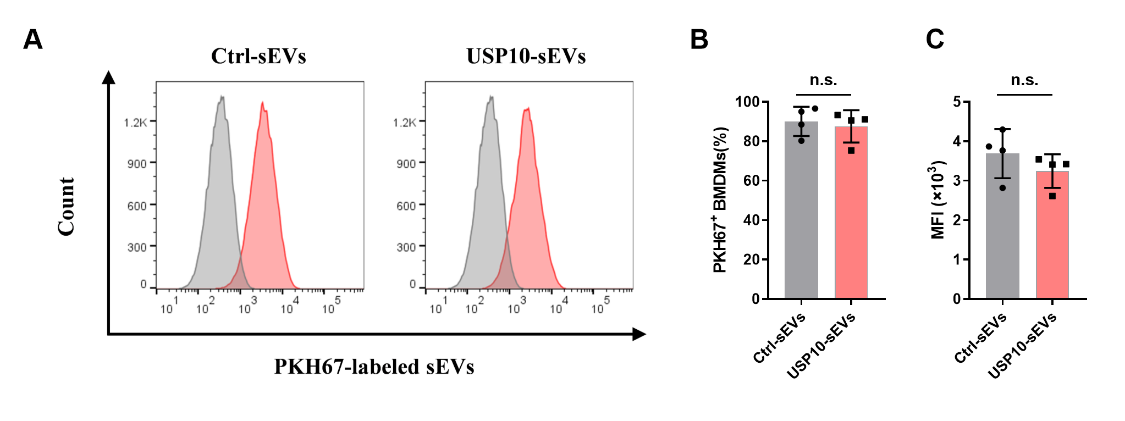


**Supplementary Figure 8. Cellular uptake of the engineered USP10-sEVs in primary bone marrow-derived macrophages (BMDMs).** A) Flow cytometry analysis. B) Quantitative analysis of the percent of PKH67^+^ BMDMs. C) Quantitative analysis of mean fluorescence intensity (MFI) of PKH67-labeled sEVs in BMDMs. Data are denoted as mean ± SD, n=4 per group; n.s. no significance.


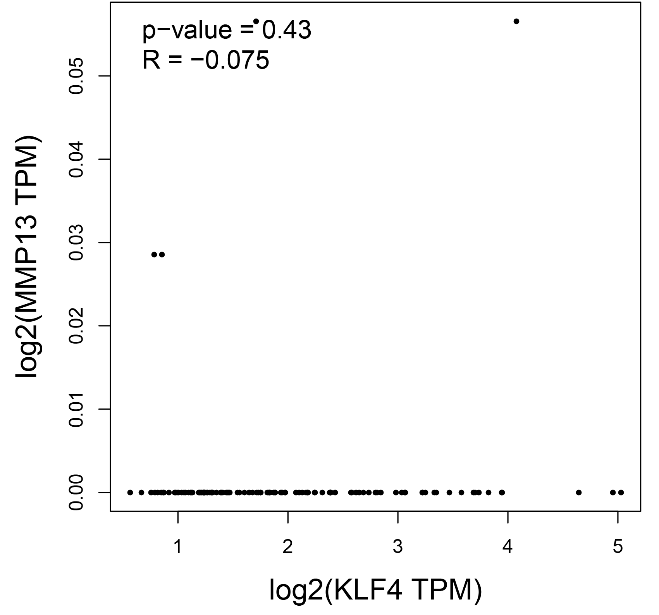


**Supplementary Figure 9. Correlation analysis between KLF4 and MMP13 in normal liver tissues from GEPIA database.**

**Supplementary Table 1. Primers used in the qRT-PCR analysis**

| **Gene** | **Forward Primer** | **Reverse Primer** |
| --- | --- | --- |
| αSMA（mouse) | CGAGCCGAGAGTAGCAGTTGTAG | AGCCATTGTCGCACACGAG |
| Col1a1（mouse) | TCTAGACATGTTCAGCTTTGTGGAC | TCTGTACGCAGGTGATTGGTG |
| Actin（mouse) | CAGCACAATGAAGATCAAGATC | CGGACTCATCGTACTCCTGCTT |
| TNFα（mouse) | GACCCTCACACTCAGATCATCT | CCTCCACTTGGTGGTTTGCT |
| IL-6（mouse) | CCACTTCACAAGTCGGAGGCTTA | GCAAGTGCATCATCGTTGTTCATAC |
| iNOS（mouse) | AGTCTCAGACATGGCTTGCCCCT | GCTGCGGGGAGCCATTTTGGT |
| CD206（mouse) | CGAGCCGAGAGTAGCAGTTGTAG | AGCCATTGTCGCACACGAG |
| IL-10（mouse) | ACTCTTCACCTGCTCCACTG | GCTATGCTGCCTGCTCTTAC |
| Arg1（mouse) | CTCCAACCCAAAGACCTTAGTG | AGGAGCAGTCATTCGGGACTTC |
| MMP2（mouse) | GATAACCTGGATGCCGTCGT | TGGTGTGCAGCGATGAAGAT |
| MMP9（mouse) | GGACCCGAAGCGGACATTG | CGTCGTCGAAATGGGCATCT |
| MMP12（mouse) | CTGCTCCCATGAATGACAGTG | AGTTGCTTCTAGCCCAAAGAAC |
| MMP13（mouse) | CTTCTTCTTGTTGAGCTGGACTC | CTGTGGAGGTCACTGTAGACT |
| USP10（mouse) | ATTTGGTCCTCAAGGGTACAGG | CGCTCTTCTGACCGTTCGTT |
| KLF4（mouse) | GAAGCGACTTCCCCCACTTCCCG | GGATGAAGCTGACGCCGAGGTG |
| USP10 (human) | CTGCCATTCTGTCCCGTCTT | CCACTGTATGGAGGAAGCTCA |
| αSMA（human) | GACAATGGCTCTGGGCTCTGTAA | TGTGCTTCGTCACCCACGTA |
| Col1a1（human) | GAGGGCAACAGCAGGTTCACTTA | TCAGCACCACCGATGTCCA |
| Actin（human) | TGGCACCCAGCACAATGAA | CTAAGTCATAGTCCGCCTAGAAGCA |

**Supplementary Table 2. Primers used for ChIP in the MMP12 promoter**

| **Name** | **Forward Primer** | **Reverse Primer** |
| --- | --- | --- |
| MMP12 Ch-IP NC | 5'CAGATTCGGCTAAAGAGTAGGC 3' | 5'TAGGCAAATAACAACTAGATG 3' |
| MMP12 Ch-IP 1 | 5'TGTAGCCCTTTTCTCCTTAC 3' | 5'GATACAAGATGCAAGGAGATA 3' |
| MMP12 Ch-IP 2 | 5'GATACAAGATGCAAGGAGATA 3' | 5'GATACAAGATGCAAGGAGATA 3' |
| MMP12 Ch-IP 3 | 5'CACGCATAAGTCACTGTCTC 3' | 5'CAGATCCAGTAGAGTTTATAGG 3' |
| MMP12 Ch-IP 4 | 5'CAGATCCAGTAGAGTTTATAGG 3' | 5'TTTCAACATACTATTTGCAAATTC 3' |
